# Evolution of the Neocortex Through RNA-Binding Proteins and Post-transcriptional Regulation

**DOI:** 10.3389/fnins.2021.803107

**Published:** 2022-01-10

**Authors:** Iva Salamon, Mladen-Roko Rasin

**Affiliations:** Department of Neuroscience and Cell Biology, Rutgers Robert Wood Johnson Medical School, The State University of New Jersey, Piscataway, NJ, United States

**Keywords:** neocortex, neurogenesis, RNA-binding proteins, post-transcriptional regulation, self-renewal, neuronal differentiation, progenitors, neuronal subtypes

## Abstract

The human neocortex is undoubtedly considered a supreme accomplishment in mammalian evolution. It features a prenatally established six-layered structure which remains plastic to the myriad of changes throughout an organism’s lifetime. A fundamental feature of neocortical evolution and development is the abundance and diversity of the progenitor cell population and their neuronal and glial progeny. These evolutionary upgrades are partially enabled due to the progenitors’ higher proliferative capacity, compartmentalization of proliferative regions, and specification of neuronal temporal identities. The driving force of these processes may be explained by temporal molecular patterning, by which progenitors have intrinsic capacity to change their competence as neocortical neurogenesis proceeds. Thus, neurogenesis can be conceptualized along two timescales of progenitors’ capacity to (1) self-renew or differentiate into basal progenitors (BPs) or neurons or (2) specify their fate into distinct neuronal and glial subtypes which participate in the formation of six-layers. Neocortical development then proceeds through sequential phases of proliferation, differentiation, neuronal migration, and maturation. Temporal molecular patterning, therefore, relies on the precise regulation of spatiotemporal gene expression. An extensive transcriptional regulatory network is accompanied by post-transcriptional regulation that is frequently mediated by the regulatory interplay between RNA-binding proteins (RBPs). RBPs exhibit important roles in every step of mRNA life cycle in any system, from splicing, polyadenylation, editing, transport, stability, localization, to translation (protein synthesis). Here, we underscore the importance of RBP functions at multiple time-restricted steps of early neurogenesis, starting from the cell fate transition of transcriptionally primed cortical progenitors. A particular emphasis will be placed on RBPs with mostly conserved but also divergent evolutionary functions in neural progenitors across different species. RBPs, when considered in the context of the fascinating process of neocortical development, deserve to be main protagonists in the story of the evolution and development of the neocortex.

## Introduction

One of the greatest innovations during the evolution of the mammalian brain is the cerebral cortex, which has arisen from the selective expansion of the dorsal telencephalon in the rostral part of the forebrain (Rakic, 2009) and manifests area-specific lamination patterns (Cadwell et al., 2019). The neocortex (neopallium or isocortex) is considered to be the most recently evolved segment of the cerebral cortex (Gilardi and Kalebic, 2021) and is thus assigned the prefix “neo”; in Latin, neocortex means “new bark” or “new cover” (Box 1). From a functional standpoint, the neocortex orchestrates complex behavioral repertoires essential to higher cognitive, motor, and sensory capabilities, including abstract thinking, metacognition, emotional intelligence, and verbal communication, all of which are well-defined abilities in primates. The selective expansion of the neocortex stems partially from both an increase in diversity and proliferative capacity of neural progenitors, which build an army of most, if not all, neuronal and glial cells (Lui et al., 2011; Gulden and Šestan, 2014; Taverna et al., 2014). As Heraclitus said: “Everything flows, and nothing abides, everything gives way, and nothing stays fixed,” this symphony of neocortical creation relies on the dynamic and irreversible flow of neurogenesis (Silbereis et al., 2016). Neurogenesis, in turn, relies on the temporal patterns of gene expression and, especially, their post-transcriptional regulation. RNA-binding proteins (RBPs) are certainly workhorses during neurogenesis; while they fill roles in neuronal maturation, morphology, synaptic connectivity, and plasticity (Keene, 2007; Darnell, 2013; Glock et al., 2017; Holt et al., 2019), these topics are outside the scope of this review. Rather, in this work, we review the recent data that illustrate the contribution of post-transcriptional regulation *via* RBPs in the modulation of progenitors’ proliferation, differentiation, and specification into neuronal and glial progeny, together with the role of RBPs in neuronal migration.

**Box 1. Evolutionary origin of the six-layered neocortex.**

It was previously thought that the six-layered neocortex arose from the simple, ancient three-layered cortices: the piriform cortex laterally and the hippocampus medially (Molnár, 2011), both of which are commonly present in mammals and reptiles (Naumann et al., 2015). This perspective was challenged by another theory that the neocortex may have evolved from the ancient reptilian telencephalon, the dorsal cortex of reptiles and the hyperpallium of birds (Glenn Northcutt and Kaas, 1995; Molnár, 2011). Tosches et al. (2018) tackled this debate by using an unbiased single-cell sequencing approach to create the neuronal subtype taxonomy of the three layered-cortex of non-avian reptiles (turtles and lizards). To track the evolution of glutamatergic and GABAergic neurons, the reptilian transcriptomic maps were compared with the transcriptomes from the mammalian ancient cortex (hippocampus) and evolutionary new six-layered neocortex. Remarkably, the study found clear homology between the reptilian three layered-cortex and the mammalian hippocampus (Tosches et al., 2018).

On the other hand, the mammalian neocortex showed an intricate mosaicism of ancient and evolutionary new neuronal subtypes. For example, the major classes of inhibitory GABAergic neurons (e.g., parvalbumin-like, somatostatin, and serotonin receptor 3A HTR3A) were detected in both mammals and reptiles, implying that the ancestor-descendant relationship was preserved. In contrast, the correlation of transcription factors specifying glutamatergic fates between reptiles and mammals showed a higher level of divergence. Since transcription factors that dictate the acquisition of upper- and lower-layer neuronal identities in mammals mutually repress each other, the authors showed clear lineage segregation in mammalian excitatory neurons. However, these upper- and lower-layer transcription factors were coexpressed in neurons of the turtle three-layered cortex, resembling the broad mammalian neuronal types. Altogether, these findings suggest that diversification of mammalian glutamatergic neurons and appearance of the six neocortical layers may have evolved from the novel repressive network that regulates these transcription factors (Tosches et al., 2018). Therefore, the neocortex appears to be an evolutionary upgrade of the reptilian three-layered neocortex, rather than an upgrade of the reptilian telencephalon.

The development of the neocortex starts with the process of neurulation (Pritz, 2005; Dugas-Ford and Ragsdale, 2015; Werner et al., 2021), during which the flat neural plate undergoes major morphological transformation to form a closed neural tube (O’Rahilly and Müller, 2006). Even though many histological traits of the neocortex are highly conserved across species (Krubitzer, 1995), a quantitative comparison of transcriptomes of the prefrontal portion of the neocortex in humans, chimpanzees, and macaques has revealed that the greatest number of differentially expressed genes (DEGs) are associated with the human neocortex. Thus, the human prefrontal cortex exhibits a unique expression profile. Notably, the DEGs are mostly related to neocortical laminar specificity (He et al., 2017). The divergence of the human neocortex from non-human primates has been further characterized by another transcriptional study conducted at the single nuclei level that compared gene expression evolution by simultaneously examining 33 different brain regions in humans, chimpanzees, macaques, and bonobos (Khrameeva et al., 2020). Only the primary and secondary cortices, limbic and association cortices, cerebellar white and gray matter, and hypothalamus exhibited large transcriptional differences in human-specific genes from non-human primates. This suggest that the aforementioned regions have undergone changes that have led to divergent evolution. Even though these studies imply that differences in transcriptional signatures among primates have contributed to the structural and functional changes that enabled the advancement of the human neocortex, the extent of these variations cannot be explained solely at the transcriptional level.

Recent findings have provided insight into how gene expression regulation, not only at transcriptional, but also at post-transcriptional (Bolognani and Perrone-Bizzozero, 2008; Alvarez-Castelao and Schuman, 2015; Gardiner et al., 2015; Popovitchenko and Rasin, 2017; Sahoo et al., 2018; Biever et al., 2019; Zahr et al., 2019; Costa et al., 2021; Hoye and Silver, 2021) and epigenetic levels (Noack and Calegari, 2018), contributes to the evolution and function of the developing neocortex. We refer the interested reader to excellent reviews that thoroughly discuss the significance of transcriptional programs during neurogenesis (Tebbenkamp et al., 2014; Andrews and Nowakowski, 2019; Miller et al., 2019; Molnár et al., 2019; García-Moreno and Molnár, 2020; Vaid and Huttner, 2020; Oproescu et al., 2021). At the post-transcriptional level, despite the fact that mRNA binding sites are less prone to genetic change than sites on chromatin (Payne et al., 2018), evolutionarily conserved RBPs exhibit intricate diversification of their developmental functions. For example, more than 1,500 RBPs have been identified in humans (Gerstberger et al., 2014). A single RBP can potentially bind to, on average, 22,000 3′ untranslated region (3′UTR)-binding sites (Van Nostrand et al., 2016), which translates into more than 33 million predicted interactions between human RBPs and targets 3′UTRs (Kim et al., 2021). In addition, the presence of highly complex regulatory interplay between the same or different RBPs can be competitive, cooperative or autoregulative in nature (Dassi, 2017), and represents another evolutionary upgrade necessary to control a wide set of mRNA targets during different stages of cortical development (genesis, migration, localization, and maturation). Moreover, post-transcriptional regulation ensures accurate acquisition of neuronal identity *via* delivery of right information regarding protein subcellular localization and abundance over time. These cell-intrinsic players, together with extrinsic factors, play an essential role in modulating the information flow from genes to proteins, thereby participating in the diversification of cell function from a fixed numbers of genes (Halbeisen et al., 2008; Ascenzi and Bony, 2017). Such coordinated regulatory activity of intrinsic and extrinsic patterns is particularly relevant to instruct the sequential flow of neocortical development (Kraushar et al., 2015; Yuzwa and Miller, 2017; Park et al., 2021a,b).

**FIGURE 1:**
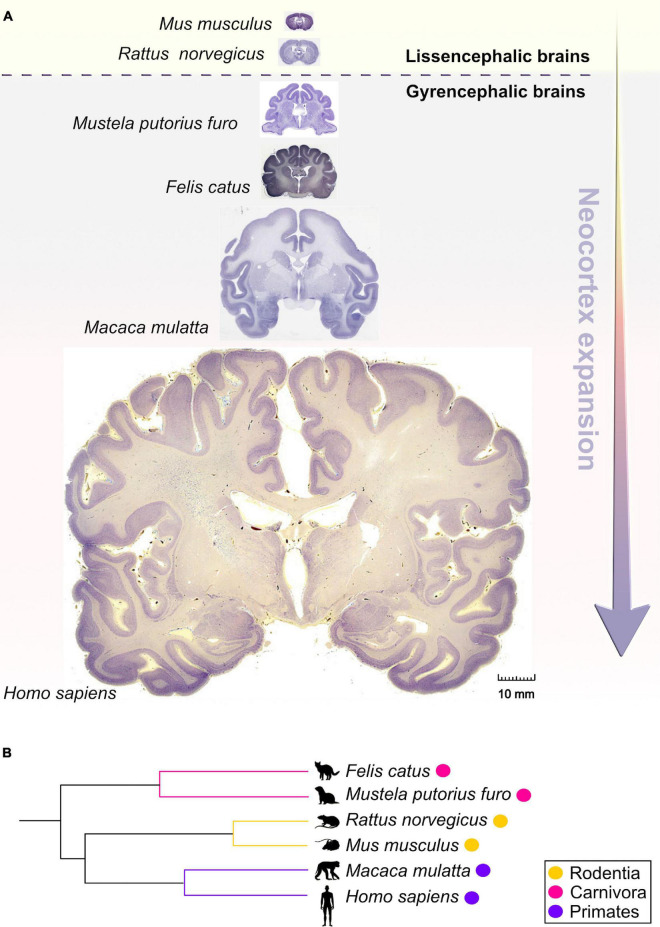
Comparative anatomy of neocortical expansion. **(A)** Nissl-stained coronal sections at the level of the anterior commissure from adult brains of *Mus musculus* (mouse), *Rattus norvegicus* (rat), *Mustela putorius furo* (ferret), *Felis catus* (cat), *Macaca mulatta* (macaque) and *Homo sapiens* (human). The arrow only illustrates the neocortical development (expansion) but does not encapsulate the evolutionary-scale relationship among these mammalian species. Mammals are grouped into lissencephalic (e.g., *Mus musculus*, *Rattus norvegicus*) and gyrencephalic species (e.g., *Mustela putorius furo*, *Felis catus*, *Macaca mulatta*, and *Homo sapiens*) based on cortical folding. Lissencephalic brains have small and smooth neocortices; the gyrencephalic brains have expanded and convoluted neocortices, with considerable variation of gyrification within and between mammalian orders. The images are scaled according to the human brain to demonstrate the notable differences in brain size and patterning of surface convolutions that have evolved from ferrets to humans (scale bar: 10 mm). Images of mouse, rat, cat, and rhesus macaque are obtained from BrainMaps next-generation atlas (Mikula et al., 2007), the ferret image was adopted from Radtke-Schuller (2018), and the human image was acquired from Michigan State University Human Brain Atlas (https://brains.anatomy.msu.edu/). **(B)** A species phylogenetic tree obtained using examples from **(A)**. This simplified representation shows that the ferrets and cats (gyrencephalic cortex) are more evolutionarily distant from humans than mouse and rats (lissencephalic cortex).

**FIGURE 2:**
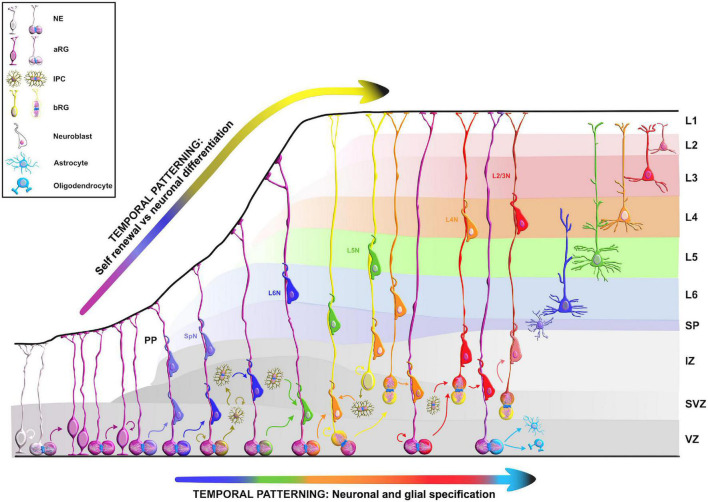
Temporal molecular patterning during neocortical development. The development of the mammalian neocortex can be conceptualized through the evolutionarily conserved mechanism of temporal molecular patterning. Molecular patterning of apical progenitors along two temporal branches provide an overview of the intrinsic processes that guide one of the two fate transitions that apical radial glia (aRG) undergo as neurogenesis proceeds: (1) “self-renewal vs. neuronal differentiation,” which gives rise to neuronal progeny either directly or indirectly through the generation of basal progenitors, and (2) “neuronal and glial specification,” which begins with the sequential production of layer-specific neuronal subtypes, and finishes with the generation of glial progeny during late stages of corticogenesis. NE, neuroepithelial cell; aRG, apical radial glia; IPC, intermediate progenitor cell; bRG, basal radial glia; PP, preplate; VZ, ventricular zone; SVZ, subventricular zone; IZ, intermediate zone; SP, subplate; SpN, subplate neuron; L1–L6, layers 1–6; L2/3N-L6N, layer 2/3 neuron-layer 6 neuron.

The wide range of post-transcriptional regulation can help to explain, at least in part, the evolutionary increase in size and complexity of the neocortex in primates, particularly humans (Figure 1) even without a significant expansion of the gene pool. Hence, it is important to uncover and understand the key regulatory RBPs guiding each step of mRNA metabolism that dictates neocorticogenesis. In this review, we explain how RBPs modulate the timely progression of neurogenesis from the perspective of progenitor temporal patterning or temporal-identity specification, a process by which an individual progenitor changes its fate to produce a succession of cell types with different identities (Kohwi and Doe, 2013). Temporal molecular patterning can further be subdivided into two parallel timescales, representing one of the two specific fates a progenitor can acquire: the general (neurogenic or neuronal) fate, and the specific-cell fate (Figure 2). The acquisition of general fate represents the situation when the progenitor stops self-amplifying and switches its fate to producing either neurogenic progenitors with restricted potency or terminally differentiated neurons. The acquisition of the specific cell fate describes a scenario when progenitors begin to differentiate into either layer-specific neuronal identities or glial cell types during the course of neurogenesis, contributing to the layering of the neocortex and neuronal and glial diversity (Kohwi and Doe, 2013; Oberst et al., 2019). Neuronal diversity may thus be pre-defined at the transition from progenitors to neurons, which is further supported by single-cell profiling of mouse progenitors and their immediate neuronal descendants at several developmental time points (Telley et al., 2019). Namely, a temporal change in progenitor’s behavior (from proliferative, neurogenic to differentiative) is dictated by the sequential activation of timed, overlapping transcriptional waves. These timed transcriptional profiles (birthmarks) correspond to the lower- or upper-layer neuronal identities are in turn transmitted from mother progenitors to daughter neurons, enabling the specification of layer-specific subtypes. Passive mother-to-daughter transmission of temporal birthmarks is probably exploited by the regulatory network at post-transcriptional levels as transcripts already present in progenitors become translated into proteins or stabilized in differentiating neurons for future actions (Telley et al., 2019).

Taken together, there is a huge potential and great need in understanding how an interaction network of RBPs acting on available transcripts and their functional heterogeneity participate not only in the preservation of progenitors’ fate, but also in the shaping and the expansion of developing neocortex.

## Post-Transcriptional Regulation, mRNAs Poised for Translation and RNA-Binding Proteins

**FIGURE 3:**
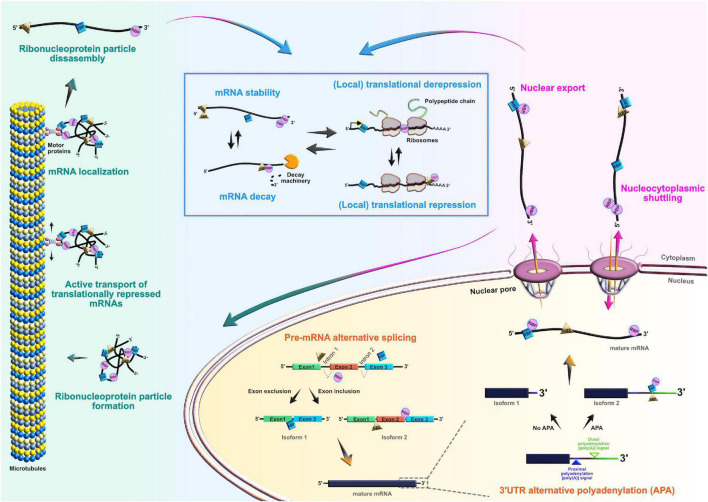
Roles of RNA-binding proteins (RBPs) in the mRNA life-cycle. At the post-transcriptional level, RBPs actively control the entire life cycle of mRNAs in both progenitors and their neuronal and glial progeny. Posttranscriptional processing begins in the nucleus, where RBPs regulate pre-mRNA alternative splicing, 3′UTR alternative polyadenylation, and nuclear export of mature mRNAs. RBPs can also act as chaperones of target mRNAs, supporting their nucleocytoplasmic shuttling. In the cytoplasm, RBPs regulate transcript localization, stability, temporal silencing, and translation, ensuring proper spatiotemporal control of protein abundance.

Transcript abundance can only partially explain exact protein levels (DeBoer et al., 2013; Kraushar et al., 2014, 2015; Popovitchenko et al., 2020), suggesting that the correlation between mRNA and protein levels depends highly on the state of the cell. In a scenario where an mRNA in developing cell is in the stable condition (steady-state level), mRNA-to-protein ratios can be predictive of each other-high mRNA levels yield high protein levels (Csárdi et al., 2015). However, this is not always the case. For example, when a neuronal cell is exposed to a changing condition, which is present during development (e.g., rapid fate and/or morphological transitions), the mRNA–protein ratio may become perturbed. As a result, transcription initiation generally may be too slow to allow the cell to confront the dynamic changes with rapid and organized agility. Indeed, it is misleading to rely solely on mRNA steady-state snapshots as a reliable proxy of protein abundance (Tahmasebi et al., 2019). This suggests that another type of regulatory network is necessary after transcription; post-transcriptional regulation provides a more precise, faster, and local reaction to various developmental demands by modifying, activating, degrading, or repressing the functional assortment of already present transcripts. These mRNA processing events, commonly known as the ribonome (Mansfield and Keene, 2009), include splicing, alternative polyadenylation, editing, stabilization, temporal silencing, targeted localization, and translation. RBPs, together with ribosomal proteins and non-coding RNAs [e.g., microRNA and long non-coding RNA (lncRNA)], are thought to be the key components of the post-transcriptional machinery since they shape the final output of the ribonome (Kwan et al., 2012; DeBoer et al., 2013; Doxakis, 2014; Mao et al., 2015; Pilaz and Silver, 2015; Kraushar et al., 2016, 2021; Ceci et al., 2021; Park et al., 2021b). The array of post-transcriptional network activities indicate that dynamic control of the transcriptome is a multifaceted series of events, necessary for the careful orchestration of the cellular behavior during neocortical development (Figure 3).

**FIGURE 4:**
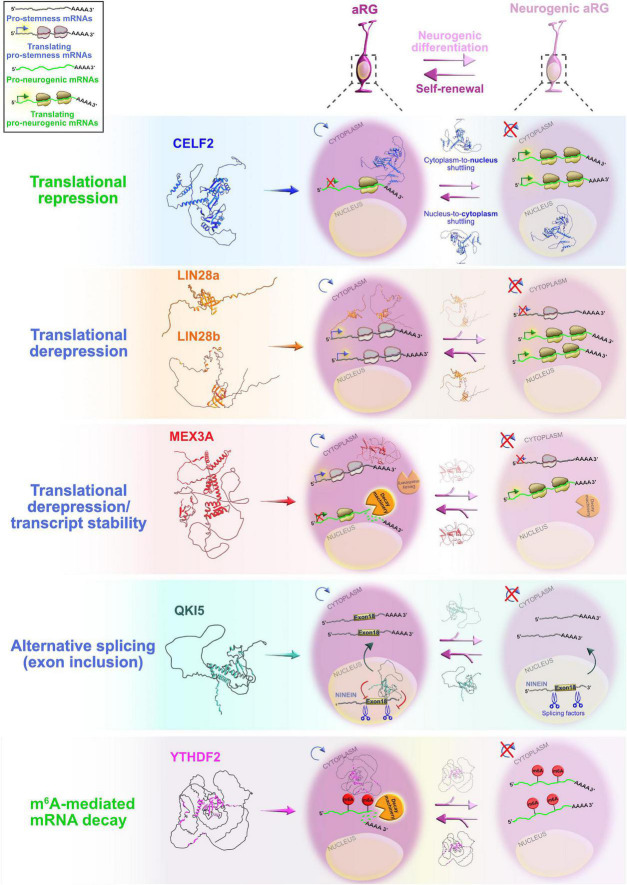
Post-transcriptional regulation by RNA-binding proteins (RBPs) and transcriptional priming in apical radial glia (aRG). Summary of the functional roles of RBPs CELF2, LIN28a/b, MEX3A, QKI5, and YTHDF2 in determining the fate of aRG, which are transcriptionally primed to differentiate into neurons. Cytoplasmic CELF2 maintains aRGs in the undifferentiated state by translationally repressing pro-neurogenic mRNAs. LIN28a/b achieves the same outcome by promoting the expression of pro-self-renewal transcripts. The regulatory mechanism by which MEX3A contributes to aRG maintenance and controls the appropriate time of aRG differentiation is unclear; MEX3A may either act as a translational repressor/derepressor of pro-neurogenic/pro-stemness mRNAs, or it can promote transcript stability/decay. The nuclear isoform of QKI (QKI5) controls the aRG-to-neuron transition *via* pre-mRNA alternative splicing (e.g., inclusion of exon 18 into *Ninein* pre-mRNA protects aRG proliferative capacity). YTHDF2 promotes N^6^-methyladenosine (m^6^A)-mediated decay of pro-neurogenic transcripts, acting as a pivotal regulator of self-renewal capabilities of aRG. The predicted tertiary and secondary full-length protein structures of RBPs in *Homo sapiens* are adopted from https://www.uniprot.org: CELF2 (O95319), LIN28a (Q9H9Z2), LIN28b (Q6ZN17), MEX3A (A1L020), QKI (Q96PU8), and YTHDF2 (Q9Y5A9).

Despite the fact that RBPs have assigned significant roles during neocortical development, it is largely unclear how each RBP contributes to neocortical development. The main challenge is to identify target mRNAs of RBPs at different developmental stages. While spatiotemporal target identification is a challenge, a subset of transcripts, which often encode functionally related proteins, can be regulated at multiple levels by virtue of binding to the same RBP or cohort of RBPs, a concept called the RNA regulon hypothesis (Keene, 2007; Morris et al., 2010). This is one way in which regulatory RBP–mRNA interactions can activate either general or cell-type specific developmental pathways during specific stages of neurogenesis. Various RBPs are already recognized as highly important for the protection of progenitors’ neurogenic potentials (Box 2), such as FMRP, Smaug2, Nanos1, Rbfox, and polypyrimidine tract-binding protein 1 (Ptbp1). Since their function during neurogenesis has been previously reviewed in detail (Pilaz and Silver, 2015; Popovitchenko and Rasin, 2017; Zahr et al., 2018, 2019; Park et al., 2021b), we will focus on deciphering the function of RBPs whose fascinating regulatory roles during early neurogenesis have recently become elucidated (Figure 4).

**Box 2. Neurogenic potential of apical progenitor cells.**

It is crucial to understand the process of neuronal production and the main steps of the prenatal neocortical development, known as cortical neurogenesis. Neuroepithelial cells (NEs) are the origin of all excitatory cortical neurons, astrocytes, and oligodendrocytes. NE form a single cell layer of primordial cells in the apical germinative or ventricular zone (VZ). Due to their polarized morphology along the apico-basal axis, NE connect the ventricular (apical) surface with the pia (basal lamina) and are linked together through the adherens junction (AJ) belt in the VZ. More importantly, NE behave as neural progenitor cells (NPCs), undergoing extensive symmetric proliferative divisions to expand the early progenitor pool (Rakic, 1995). Ultimately, their self-amplifying capacity will enable the expansion of the neocortex in both lateral and radial dimensions by influencing the number of neurons generated. NE undergo interkinetic nuclear migration (INM), which is necessary for optimal usage of the limited ventricular surface available for division.

During INM, the positioning of the NE nucleus along the apico-basal axis in the VZ corresponds to stages of the cell cycle. When the nucleus is further from (when in G1-, S-, G2-phase) or closer to (when in M phase) the ventricular surface, the result is a pseudostratified conformation of NE in the VZ (Takahashi et al., 1995; Florio and Huttner, 2014).

During early phases of mammalian neocortical development, NE switch to asymmetric consumptive cell divisions to differentiate into another type of apical NPC, called the apical or ventricular radial glia cells (aRG). This event at the early stages of development signals the beginning of the neurogenic phase where at least one daughter cell stops dividing by becoming a neuron, thereby balancing the ratio between proliferation and differentiation (Noctor et al., 2001; Shitamukai and Matsuzaki, 2012). aRG serve two main functions during neocortical development. Firstly, as indicated in their name, glia, which originates from the Greek word “glía” and translates into glue, aRG act as a scaffold guiding the migration of early newborn neurons from their place of birth to their destined position in the neocortex (Kriegstein et al., 2006). Just like NE, aRG are attached to the VZ by their apical endfeets and project their basal processes directly to the pial surface (basal lamina). Secondly, aRG also express neuroepithelium properties by retaining INM capacity, even though their proliferative potential is more restricted than NE (Uzquiano et al., 2018). aRG can either self-renew through a series of proliferative symmetric divisions, or divide asymmetrically in a proliferative and consumptive manner. Asymmetric proliferative division generates one daughter cell that is identical to its mother aRG, and another daughter cell that is either an immature postmitotic neuron (*direct neurogenesis*), or one of the two main types of more committed BPs: (1) transit-amplifying progenitors or intermediate progenitor cells (IPCs), or (2) outer radial glial cells or basal radial glia (bRG) (*indirect neurogenesis*) (Kriegstein et al., 2006; Lui et al., 2011; Xing et al., 2021). As a result of this enormous accumulation of BP, the neocortex becomes even thicker and is comprised of distinct developmental regions: the VZ, the subventricular zone (SVZ), the intermediate zone (IZ), the subplate (SP), the cortical plate (CP), and the marginal zone (MZ).

### Embryonic Lethal, Abnormal Vision-Like and CUGBP, ELAVL-Like Family

**FIGURE 5:**
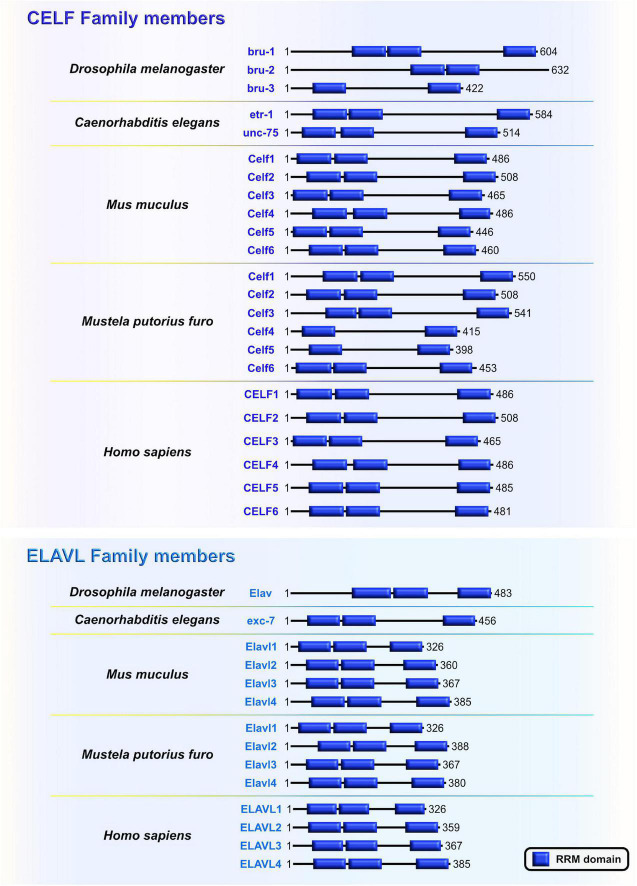
The CUGBP, ELAVL-like family (CELF) and embryonic lethal, abnormal vision-like (ELAVL) family members are closely related. Similarities and differences of RNA-binding domain, RNA Recognition Motif (RRM; blue) which is present in evolutionarily conserved RNA-binding proteins (RBPs) CELF and ELAVL in *Drosophila melanogaster*, *Caenorhabditis elegans*, *Mus musculus*, *Mustela putorius furo*, and *Homo sapiens* according to UniProt database (https://www.uniprot.org) and NCBI (https://www.ncbi.nlm.nih.gov/). The UniProtKB of NCBI accession numbers are indicated below for each member of CELF and ELAVL family in *D. melanogaster*: Bruno (bru)-1 (Q960Z4), bru-2 (Q7K108), bru-3 (Q9VU91), and Embryonic Lethal, Abnormal Vision (Elav) (P16914); *C. elegans*: ELAV-Type RNA binding-protein family (etr)-1 (G5EF03), uncoordinated (unc)-75 (G5EE68), and excretory canal abnormal (exc)-7 (Q20084); *M. musculus*: Celf1 (P28659), Celf2 (Q9Z0H4), Celf3 (Q8CIN6), Celf4 (Q7TSY6), Celf5 (A0A5F8MPH2), Celf6 (Q7TN33), and Elavl1 (P70372), Elavl2 (Q60899), Elavl3 (Q60900), Elavl4 (Q61701); *M. putorius furo*: Celf1 (M3XXX8), Celf2 (M3YY92), Celf3 (M3XWY8), Celf4 (M3XPL9), Celf5 (M3XX93), Celf6 (XP_004758414.1), and Elavl1 (M3Y9C6), Elavl2 (M3YX03), Elavl3 (M3Y100), Elavl4 (M3Y730); and *H. sapiens*: CELF1 (Q92879), CELF2 (O95319), CELF3 (Q5SZQ8), CELF4 (Q9BZC1), CELF5 (Q8N6W0), CELF6 (Q96J87), and ELAVL1 (Q15717), ELAVL2 (Q12926), ELAVL3 (Q14576), ELAVL4 (P26378).

Embryonic lethal, abnormal vision-like (ELAVL) and CUGBP, ELAVL-like family (CELF) proteins belong to evolutionarily conserved, yet distinct, families of RBPs that display similar domain structures containing two N-terminal RNA recognition motifs (RRMs) (RRM1 and RRM2) followed by a divergent linker domain and a third C-terminal RRM3 (Ladd et al., 2001; Figure 5). In mammals, the four members of ELAVL family (ELAVL1 or HuA/R, ELAVL2 or HuB, ELAVL3 or HuC, and ELAVL4 or HuD) are abundantly present in neurons. An exception is the ubiquitously expressed ELAVL1 (Mirisis and Carew, 2019). By binding to the AU-rich elements in the 3′UTRs of their mRNA targets, ELAVL proteins play a pivotal role in the post-transcriptional regulatory network during neocortical development and postnatal plasticity (Bolognani et al., 2010; Ince-Dunn et al., 2012; Dougherty et al., 2013; Perrone-Bizzozero, 2013; DeBoer et al., 2014; Kraushar et al., 2014; Suhl et al., 2015; Wang et al., 2015; Dell’Orco et al., 2020; Sena et al., 2021). In human and rodents, the CELF family has six different proteins (CELF1–6) that have the capacity to shuttle between the nucleus and cytoplasm to modulate various aspects of mRNA metabolism at the post-transcriptional level by binding GU-rich elements in the transcripts (Gallo and Spickett, 2010). When compared to other CELF members, CELF1 and CELF2 are phylogenetically clustered together due to the highest level of structural topology and an overlapping, ubiquitous expression pattern. In contrast, CELF3–6 have more restricted expression, primarily in the nervous system (Dasgupta and Ladd, 2012).

The CELF and ELAVL families are linked to neural development and as such the polymorphisms in *CELF* and *ELAVL* genes, as well as alterations in the functional properties of their respective proteins, are associated with neurodevelopmental disorders (Popovitchenko et al., 2020). For example, Itai et al. (2021) have identified for the first-time that heterozygous *CELF2* mutations in unrelated individuals resulted in a range of overlapping clinical symptoms. These symptoms include neurodevelopmental and epileptic encephalopathy, intellectual disability, and autistic behavior – all to varying severity. This suggests that *CELF2*, and specifically its dosage, is critical to normal neuronal function (Itai et al., 2021). Another recent study has corroborated previous findings by identifying additional *de novo* heterozygous missense *CELF2* mutations in RRM3 in patients with neurodevelopmental defects and cortical malformations. In addition, authors observed that CELF2 exhibits bipartite compartmentalization in mouse embryonic day 15 (E15) neocortices: while cytoplasmic expression is dominant in apical radial glia (aRG), nuclear localization is mainly present in IPCs (IPCs) and newborn neurons (MacPherson et al., 2021). Using a well-designed experimental setup with *in vivo* and *in vitro* experiments, the findings point to a mechanism by which cytoplasmic–nuclear shuttling of CELF2 serves as a translational repression–derepression switch between self-renewal and differentiation programs of aRG. Specifically, the authors revealed that cytoplasmic CELF2 binds and recruits proneural factors (such as *Neurog2*, *Neurod1*, and *Tbr2*) and neurodevelopmental disease-associated mRNAs into processing bodies for translational repression, thereby maintaining NPC identity and controlling the NPC fate decision (MacPherson et al., 2021). Itai et al. (2021) also noticed an aberrant cytoplasmic accumulation of CELF2 after transfecting human HEK293T cells and African green monkey COS7 cells with plasmids containing disease-associated missense and frameshift variants. These results confirm the necessity of post-transcriptional regulation, and specifically of cytoplasmic–nuclear shuttling activity of CELF2, for the maintenance of progenitor self-renewal properties.

**FIGURE 6:**
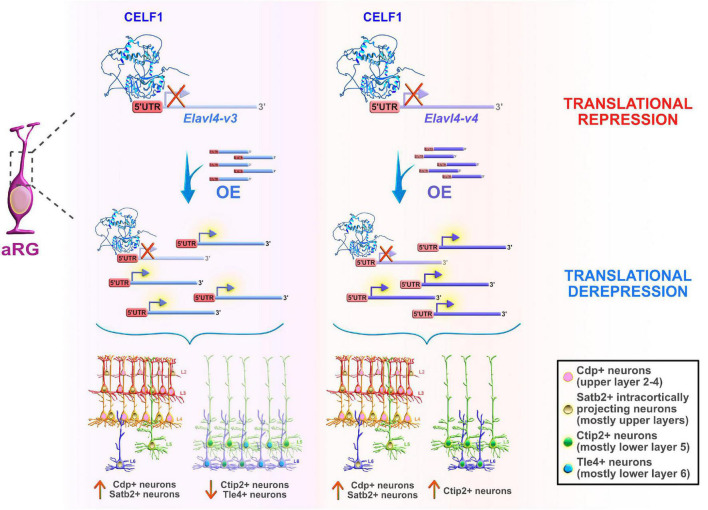
Celf1 translationally regulates Elavl4 to dictate the development of glutamatergic neuronal subtypes. RNA-binding protein (RBP) Celf1 operates as an isoform-specific translational repressor of RBP Elavl4 by binding to its 5′UTRs in apical radial glia (aRG). Translational derepression of Elavl4 isoforms (Elavl4-v3 and Elavl4-v4) affected the production of specific neuronal laminar identities as identified using transcription factor profiling (e.g., transcription factors Cdp, Satb2, Ctip2, and Tle4 are associated with distinct neuronal subtypes in the neocortex). The conditional overexpression (OE) of 5′UTR Elavl4-v3 in aRG increased the number of upper layer Cdp-positive (+) and intracortically projecting Satb2+ neuronal subtypes, while the neuronal density of lower layer Ctip2+ and Tle4+ identities decreased. The conditional 5′UTR Elavl4-v4 OE in aRG positively influenced the production of both upper layer (Cdp+ and Satb2+) neuronal identities and specific subtype of lower layer (Ctip2+) neurons. These results highlight the importance of studying RBP–RBP interactions to decipher the mechanisms underlying the extraordinary diversity of neuronal and non-neuronal cell types in the developing neocortex. The predicted tertiary and secondary full-length protein structure of CELF1 (Q92879) in *Homo sapiens* is adopted from https://www.uniprot.org.

On the other hand, another Celf member, Celf1, was found to regulate the specification of neocortical neuronal identities during neurogenesis (Popovitchenko et al., 2020). The study showed that only one of two Celf1 isoforms (Celf1 short, Celf1S) binds the 5′UTRs of specific isoforms of the RBP *Elavl4* (*HuD*, *-v3*, and *-v1&4*) to induce translational repression in aRG during early stages of neurogenesis. Not surprisingly, Celf1 and its downstream target *Elavl4* have opposite protein expression patterns in both human and mouse neocortical progenitors. The expression of Celf1 radically decreases in the aRG of VZ, and dramatically rises in the CP from early to later stages of neurogenesis. In contrast, Elavl4 (*-v1&4*) is expressed only in the post-mitotic neurons in CP early in development, but its presence becomes noticeable in VZ (*-v3*), and even more obvious in the IZ and CP (*-v1&4*) at later neurodevelopmental stages (Popovitchenko et al., 2020), while corresponding mRNAs are expressed at steady-state across stages. Another single-cell sequencing study also showed that *Elavl4* mRNA levels are upregulated in human intermediate progenitors that have a high capacity to differentiate into early neurons during neurogenesis (Pollen et al., 2015). Silencing of *Celf1* in mouse aRG, in which Elavl4 protein synthesis is then regularly derepressed, favored the acquisition of upper layer neuronal identities, at the expense of lower layer neuronal subtypes, and appeared to impair axonal projections reaching the striatum. On the other hand, Cefl1S overexpression (OE) experiments resulted in a reduced number of upper layer neuronal subtypes and ipsilateral atypical accumulation of axonal tracts that should have passed the corpus callosum. Similarly, OE of either *Elavl4-v3* and *-v4* with their 5′UTRs in mouse aRG promoted the acquisition of the upper layer neuronal identities but exerted opposing effects on the acquisition of the lower layer neuronal subpopulations (Figure 6). Thus, Celf1-guided translational repression of Elavl4 isoforms is a key element in determining the balanced development of upper and lower neuronal identities, and also in the establishment of the proper neuronal connectivity during mouse and potentially human development (Popovitchenko et al., 2020).

To explore the mechanism underlying the directed migration of neurons, a recent study used *Caenorhabditis elegans* and implicated etr-1, a Celf1 homolog, in the regulation of long-range migration of the Q neuroblast lineage neurons (AQR and PQR) in nematode larvae (Ochs et al., 2020). A forward genetic approach identified a mutation in *etr-1(lq61)* that is responsible for the migratory defects of AQR and PQR neurons; the *etr-1(lq61)* mutation is hypomorphic in nature since it induces the premature stop codon in the *etr-1* gene. In contrast, silencing of *etr-1* in *C. elegans* causes embryonic lethality and body wall muscle defects, corroborating previous findings of mouse neonatal lethality due to global *Celf1* deletion (Kress et al., 2007; Cibois et al., 2012; Popovitchenko et al., 2020). Both muscle-specific CRISPR/Cas9 genome editing, and *etr-1* expression driven only by the body-wall-muscle specific promoter were able to rescue the migratory phenotype. These findings showed that etr-1 influences neuronal migration in a non-autonomous manner from body wall muscle, interacting directly or indirectly with the Wnt pathway to generate external factors that modulate AQR and PQR migration (Ochs et al., 2020). However, the question of whether etr-1 translationally regulates its mRNA targets in muscles, and if these targets are shared with mammalian Celf1 remains to be addressed.

Unlike the regulatory roles of Celf2 and Celf1/Elavl4 in neural generation and specification of glutamatergic excitatory neurons, Elavl3 (HuC) was recently implicated in the differentiation of GABAergic inhibitory neurons by participating in alternative cleavage and polyadenylation, which strongly influence 3′UTR usage during embryonic neuronal differentiation (Wamsley et al., 2018; Grassi et al., 2019). Authors used adherent neural stem cells (ANS) derived from mouse E14 embryonic forebrain that can easily and efficiently differentiate toward an inhibitory lineage. Results confirmed previous findings that transcripts preferentially chose widespread lengthening of their 3′UTRs when the progenitors were undergoing differentiation (Ji et al., 2009). Elavl3 appears to act as a master regulator in 3′UTR-alternative polyadenylation selection; indeed, silencing of Elavl3 in differentiating inhibitory neurons resulted in the preferential usage of the shorter 3′UTR options. This resulted in the downregulation of neural-associated transcripts (such as *Tubβ3* and *Gad1*). Such events indicate aberrations in the differentiation process. Interestingly, among all Elavl family members, only Elavl4 was significantly downregulated in the states of reduced proliferation and early stages of differentiation (Grassi et al., 2019), supporting its increased expression at later stages during neocortical development (Popovitchenko et al., 2020). Since *Elavl3* and *Elavl4* share a high degree of sequence homology, it would be interesting to investigate if Elavl4 plays the same role in alternative polyadenylation-driven differentiation of glutamatergic neurons, and whether Celf1 translationally represses *Elavl3* in proliferating progenitors during neocortical development.

### Lineage Abnormal 28

**FIGURE 7:**
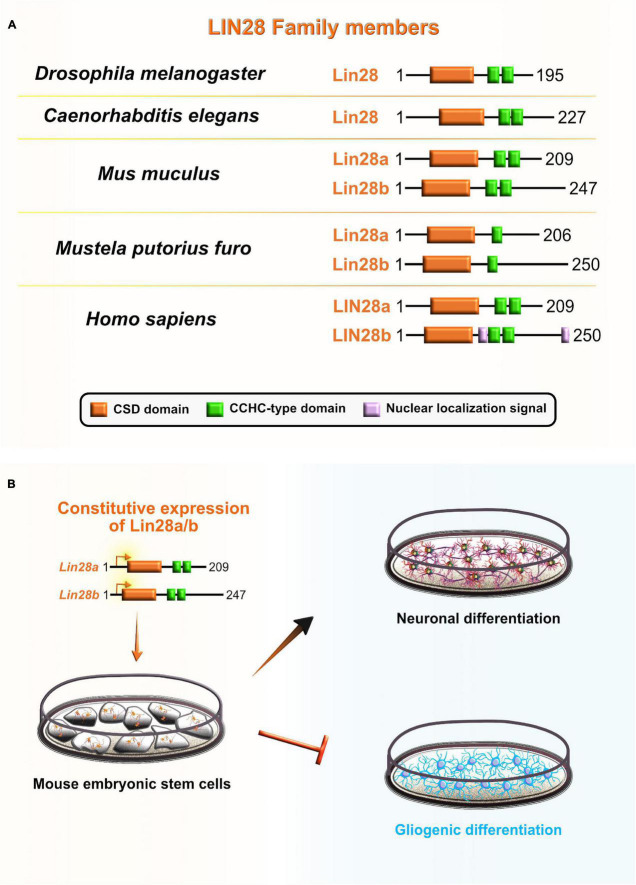
Role of lineage abnormal 28 (LIN28) during neocortical development. **(A)** Schematic presentation of structural domains of evolutionarily conserved RNA-binding protein LIN28 in *Drosophila melanogaster*, *Caenorhabditis elegans*, *Mus musculus*, *Mustela putorius furo*, and *Homo sapiens* as per UniProt database (https://www.uniprot.org). Different domains are represented as colored boxes, also in order from left to right: cold shock domain (CSD; orange), CCHC type zinc finger domains (green), nuclear localization signal motif (rose). The UniProtKB accession numbers are indicated below for each member of LIN28 family in *D. melanogaster*: cell lineage abnormal 28 (Lin28) (Q9VRN5); *C. elegans*: Lin28 (P92186); *M. musculus*: Lin28a (Q8K3Y3), Lin28b (Q45KJ6); *M. putorius furo*: Lin28a (M3YWA5), Lin28b (M3YDK6); and *H. sapiens*: LIN28a (Q9H9Z2), LIN28b (Q6ZN17). **(B)** Lin28 is expressed at high levels during early neocortical development. These levels rapidly decrease at later stages of neurogenesis to allow for the sequential generation of neuronal and glial fates. The constitutive expression of Lin28 in undifferentiated stem cells switches off the generation of glial cell fates while supporting the establishment of neuronal fates.

Cell lineage abnormal 28 (Lin28) is an RBP that acts as a major translational reprogramming factor (Zhang et al., 2016) and has a unique pairing of two RNA-binding domains: the N-terminal cold shock domain (CSD) and two CCHC type zinc finger domains, with the former resembling an RRM domain that exclusively binds RNA and the latter two which participate in the binding of RNA and also DNA (Figure 7A). Both binding domains are highly conserved across species: including worms, flies, frogs, mice, and humans (Moss and Tang, 2003; Faas et al., 2013). Vertebrates have two Lin28 paralogs that have high sequence similarity: Lin28a and Lin28b (Mayr and Heinemann, 2013). Due to their unique bipartite structures, Lin28 acts as a master regulator of both miRNAs and mRNAs by inhibiting the biogenesis of *let-7* family miRNAs and directly modulating the translation of specific cohort of mRNAs (Wilbert et al., 2012; Ustianenko et al., 2018). The conservation of Lin28 on-early/off-late expression profile in the neocortex supports its indispensable role during embryonic development. Lin28a/b are highly abundant during early stages of neocortical development, especially in NE and aRG, while their expression gradually decreases when neuronal differentiation dominates over progenitor proliferation (Moss and Tang, 2003; Yang et al., 2015).

Consistent with its distinct expression pattern, Lin28 was found to be one of the first heterochronic regulators of cell fate in *C. elegans* larvae, in which *Lin28* loss-of-function causes precocious maturation of hypodermal seam cells due to the absence of progenitors’ symmetric divisions (Ambros and Horvitz, 1984). In contrast, *Lin28* OE at the second larval stage causes enormous proliferations due to the reiterations of progenitors’ symmetric divisions (Moss et al., 1997). Similarly, Yang et al. (2015) showed that Lin28 paralogs are required for the maintenance of the cell-cycle progression and mitotic entry in mouse embryos, which are in turn necessary for the sustained proliferation of progenitors during neocortical development. The deletion of *Lin28a* in mouse embryos results in the significant reduction of both aRG and IPC, as reflected in the appearance of mild microcephaly (Yang et al., 2015). This suggest that RBP dysfunction during neocortical development can cause severe neurodevelopmental disorders (Kraushar et al., 2014; Mao et al., 2015).

On the other hand, *Lin28b* knockout (KO) embryos do not exhibit any cellular or morphological phenotypes reminiscent of the ones observed in *Lin28a* KOs (Shinoda et al., 2013; Herrlinger et al., 2019). Mouse embryos that lack one allele of *Lin28b* in *Lin28a* KO background exhibit a more severe developmental phenotype, suggesting that Lin28a/b have both essential and partially redundant functions during neocortical development (Yang et al., 2015). Furthermore, double deletion of *Lin28a/b* in mouse embryos caused the most deleterious morphological phenotype: neural tube defects and embryonic lethality. Such developmental consequences are attributed to the reduced proliferation of NE and premature neuronal differentiation. This indicates that Lin28a/b stimulate the symmetric divisions of apical progenitors required for normal neural tube closure, but are not necessary to trigger the neuronal differentiation programs that arise later during development (Herrlinger et al., 2019). Hence, Lin28a/b are fundamentally important for progenitors’ self-renewal capacity by maintaining the threshold levels that control the transition of apical progenitors from symmetric to asymmetric divisions. This is consistent with the finding that *Lin28a* OE in mouse embryos causes excessive aRG amplification by preventing their cell-cycle exit, and concurrently affecting their conversion to IPC at the advantage of neuronal production (Yang et al., 2015).

Yang et al. (2015) also showed that Lin28 regulates the stemness of apical progenitors through the *let-7* independent mechanism by acting as a translational regulator of a subset of mRNAs, including *Hmga2*, *Igf2*, *Igf1r*, *Akt1/3*, and *Imp1*. These mRNA targets are mostly involved with the Igf2–mTOR signaling pathway that drives progenitor proliferation (Hentges et al., 2001). Other studies have directly linked Lin28a function with translational regulation and cell division by showing that *Lin28a* silencing decreases the levels of its targets *Hmga2* and *Igf1r*, whereas *Lin28a* OE upregulates *Hmga2* and *Igf2* in mouse primary cultures of electroporated cortical neurons (Bhuiyan et al., 2013; Jang et al., 2019). Polysome profiling analysis of *Lin28a/b* KO embryonic neocortices indicated that transcripts associated with translation, ribosome biogenesis, mTOR pathway, and cell cycle are decreased, whereas transcripts involved with neuronal differentiation are significantly upregulated in double mutants. Mutant phenotype (macrocephaly and an abnormal number of apical progenitors) can be rescued by the ribosomal protein L24 hypomorphic allele in the background of *Lin28a* OE mouse line, suggesting that Lin28 mainly acts as a translational derepressor in the apical progenitors during early neurogenesis (Herrlinger et al., 2019).

Several lines of evidence also suggest that Lin28 may be involved in the regulation of temporal-identity specification. To gain better insight into the role that Lin28 plays in neurogliogenesis, during which Lin28 levels are rapidly reduced, Balzer et al. (2010) constitutively expressed *Lin28* in differentiating mouse embryonic carcinoma cells. The authors noticed that progression of neuron-to-glia cell fate was severely affected, evidenced by increased neurogenesis and decreased gliogenesis. This suggests that Lin28 blocks astroglial differentiation programs and preferentially promotes the neuronal-lineage transition in progenitors (Balzer et al., 2010; Figure 7B). Another recent study *in vitro* confirmed that Lin28 controls the neurogliogenic decision independently of the *let-7* mechanism. Namely, *Lin28a/b* OE in mouse ESC increased the Yap1 protein levels, whereas the inhibition of Yap1 in Lin28a/b OE cells partially rescued the glial differentiation defect. Lin28a/b directly binds and translationally regulates *Yap1* mRNA, which seems to be an important regulatory mechanism in controlling the cell-fate switch toward astrogliogenesis (Luo et al., 2021). These findings show that Lin28 function in sequential progression of cell fate is conserved between *C. elegans* and mammals, and specifically through post-transcriptional control in both.

### Muscle Excess 3

**FIGURE 8:**
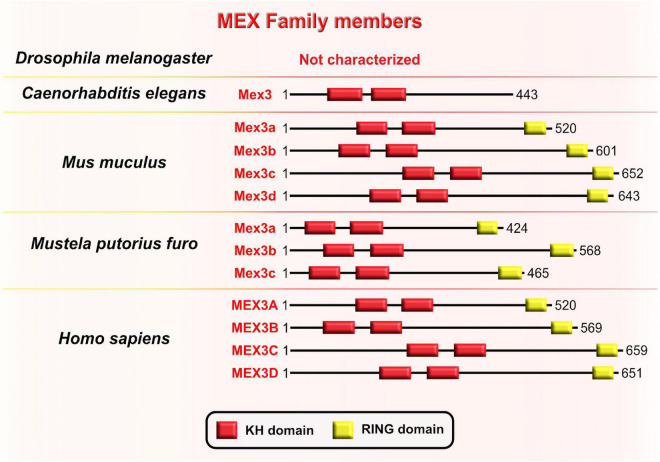
Muscle excess 3 (Mex3) family of evolutionarily conserved RNA-binding proteins. Schematic presentation of binding domains K homology (KH) domain (red), and RING domain (yellow) in Mex3 family members in *Drosophila melanogaster*, *Caenorhabditis elegans*, *Mus musculus*, *Mustela putorius furo*, and *Homo sapiens* adopted by either UniProt database (https://www.uniprot.org) or NCBI (https://www.ncbi.nlm.nih.gov/). The UniProtKB of NCBI accession numbers are indicated below for members of Mex3 family in *C. elegans*: Mex3 (H2L067); *M. musculus*: Mex3a (NP_001025061.2), Mex3b (Q69Z36), Mex3c (Q05A36), Mex3d (Q3UE17); *M. putorius furo*: Mex3a (XP_012904401.1), Mex3b (XP_004763707.1), Mex3c (XP_012904006.1); and *H. sapiens*: MEX3A (A1L020), MEX3B (Q6ZN04) MEX3C (Q5U5Q3), MEX3D (Q86XN8).

Muscle excess 3 (Mex3) was first discovered in *C. elegans* where it is required for the maintenance of germline totipotency. This RBP is characterized by two K homology (KH) domains and has nucleocytoplasmic shuttling ability (Draper et al., 1996; Figure 8). By binding to their targets’ 3′UTRs *via* conserved KH-domains, Mex3 acts as both a translational repressor and as a key regulator of the asymmetric expression of transcripts encoding critical cell fate determinants. One such transcript is the maternally supplied transcript Pal-1 (CDX1 homolog) which promotes specification of either hypodermal or muscle precursors during embryogenesis in worms (Edgar et al., 2001). The asymmetric distribution of maternal transcripts in early blastomeres serves as a base for proper patterning of nematode embryos. An observed phenotype in nematode embryos with mutated Mex3 was the irregular production of body-wall muscles and hypodermal cells from the anterior founder cell, hence the name “muscle excess.” The developmental pattern characteristic for the posterior germline lineage of the wild-type embryo was detected in the anterior blastomere of the Mex3 mutant embryos (Draper et al., 1996). Mex3 not only links cell polarity to the specification of cell fates in nematode embryos, but also plays a redundant role with other RBPs to promote mitotic proliferations of germline stem cells in adult nematodes (Ariz et al., 2009; Pagano et al., 2009).

In the sea urchin *Paracentrotus lividus*, the homologous protein to the Mex3 is named RING finger and KH-domain (RKHD); it is also maternally supplied and strongly expressed during early zygotic development (Röttinger et al., 2006). The fact that RKHD is highly recruited onto polysomes after fertilization additionally supports its role in the regulation of mRNA metabolism during the egg-to-embryo transition (Chassé et al., 2018). Conserved KH domains with RNA-binding capacity are present in four types of Mex3 orthologs in vertebrates (Mex3A–D) and are highly similar to Mex3 in nematodes, bolstering evolutionary conservation of its function between invertebrates and vertebrates (Pagano et al., 2009). Even though the RING domain is not a part of the Mex3 structure in nematodes, its acquisition in vertebrates is required for control of gene expression at the post-translational level through ubiquitin E3 ligase activity (Buchet-Poyau et al., 2007; Bufalieri et al., 2020). Evolutionary diversification of the Mex3 gene from nematode to mammals is reflected in the progression of its function by which Mex3 initially acts as a translational repressor in the nematode lineage and progressively gains additional ubiquitin E3 ligase activity that is required for protein degradation. It is unknown, however, whether Mex3 can regulate developmental processes post-translationally by acting as E3 ubiquitin ligase.

A Mex3A homolog was first identified as a potential regulator of adult neurogenesis in *Nothobranchius furzeri*, or killifish, which is a powerful vertebrate model to study age-related changes. *In situ* hybridization data showed that Mex3A has high expression in neurogenic niches of zebrafish embryos and young *N. furzeri* animals, which exponentially decrease with age (Baumgart et al., 2014). For the first time, the same group revealed that Mex3A indeed plays a role in embryonic vertebrate nervous system development using *Xenopus laevis* as a model system. Silencing, OE, and phenotypic rescue experiments in *X. laevis* showed that Mex3A disables neuronal differentiation during neurogenesis by maintaining neural progenitors in an undifferentiated, proliferative state. The proposed mechanism of Mex3A regulation takes place through the induction of *Sox2* and *Musashi-1* expression, both of which support the self-renewal of neural progenitors, and a simultaneous downregulation of elrC (Elavl3 homolog), which is commonly used as an early marker of neuronal differentiation. Furthermore, *in situ* hybridization of mouse embryos at E18 showed intense Mex3A signal in the proliferative regions of the VZ and SVZ, suggesting the conserved function of Mex3A in the maintenance of progenitors’ stemness competence (Naef et al., 2020). Mex3A seems to be an important post-transcriptional regulator during neocortical development, but the exact mechanism by which Mex3A regulates its targets remains elusive. The future studies should clarify whether Mex3A operates as a translational derepressor/repressor of pro-neurogenic transcripts/pro-neuronal transcripts, or if the underlying mechanism goes through the stabilization/degradation of its target transcripts.

Even though the exact role of MEX3A in human prenatal neurogenesis is yet to be uncovered, its regulation of a stemness state seems to be a recurrent topic within the human MEX3 family members. For example, *MEX3A* OE in human gastrointestinal 2D and 3D cultures strongly represses the expression of the *CDX2* intestinal transcriptional factor (Pereira et al., 2013), which functions as both a lineage-specific transcriptional enhancer of trophectoderm genes and a repressor of inner cell mass pluripotency genes during early embryonic development (Jedrusik et al., 2008; Huang et al., 2017). The binding of MEX3A to 3′UTRs of *CDX2* results in a reduction of differentiation and polarity features, which might be the turning point that enables a permissive environment for the maintenance of stemness (Pereira et al., 2013). The BrainSpan Atlas of the developing human brain (Miller et al., 2014) provides a comprehensive transcriptome map across the key stages of human development. In BrainSpain, transcripts of MEX3 homologs show the highest expression profile during the earliest embryonic stages and their expression gradually decreases toward the postnatal stages, implying that MEX3 might indeed regulate the stemness/differentiation decision during human embryonic development.

### Quaking

**FIGURE 9:**
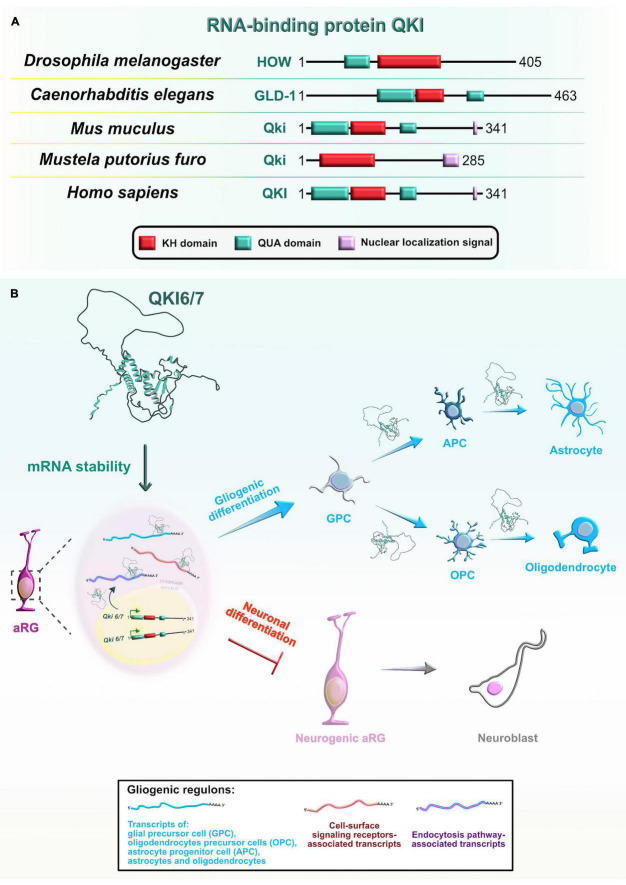
Evolutionarily conserved RNA-binding protein Quaking (Qki) and its role during later stages of neocortical development. **(A)** Domain structures of Qki in *Drosophila melanogaster*, *Caenorhabditis elegans*, *Mus musculus*, *Mustela putorius furo*, and *Homo sapiens* according to https://www.uniprot.org or https://www.ncbi.nlm.nih.gov/. The RNA-binding motif K homology (KH) domain is colored in red, Quaking (QUA; QUA1 involved in homodimerization and QUA2 involved in RNA binding) domains are labeled in cyan, and nuclear localization signal motif is in rose. The UniProtKB of NCBI accession numbers are indicated below for Qki protein in *D. melanogaster*: held out wings (HOW) (NP_524447.2); *C. elegans*: Germline Defective-1 (GLD-1) (Q17339), *M. musculus*: Qki (Q9QYS9); *M. putorius furo*: Qki (XP_004769624.1); and *H. sapiens*: QKI (Q96PU8). **(B)** Qki is selectively expressed in proliferative regions in the developing neocortex where it promotes the cell fate switch from neurons toward glial cells. Qki synergistically controls the expression of gliogenic regulons (shown in box at bottom center) by binding targets’ 3′UTRs and stabilizing target mRNAs associated with glia-, astrocyte-, oligodendrocyte precursor-, astrocyte precursor-, oligodendrocyte-, and astrocyte-specific genes, as well as cell-surface signaling receptors, and endocytosis pathway genes.

Mammalian Quaking (QKI) is another RBP with a KH-type domain (Figure 9A) that has three major spliced isoforms which differ only in their C-terminal tail: nuclear Qki5, nuclear and cytoplasmic Qki6, and predominantly cytoplasmic Qki7 (Fagg et al., 2017). The protein expression profile of two of the isoforms, Qki5 and Qki6, shows cell-type and subcellular localization specificity in the VZ during early mouse neocortical development. Even though both isoforms are exclusively coexpressed in aRG during the earliest stages of embryonic neurogenesis, their abundance rapidly decreases in IPC (Hardy et al., 1996; Wu et al., 1999; Hayakawa-Yano et al., 2017). These findings imply that Qki5 and Qki6 play an important role in modulating the progenitor proliferative state during neurogenesis.

Similarly, the evolutionary orthologs of mammalian Qki from other species, held out wings (HOW) in *Drosophila melanogaster* and germline defective-1 (GLD-1) in *Caenorhabditis elegans*, play crucial roles during embryogenesis. In *Drosophila* embryos, one of two known isoforms, HOW(L), transiently blocks cell-cycle progression to enable mesoderm invagination during the beginning of gastrulation. Mechanistically, this isoform promotes the degradation of string/Cdc25 transcripts, known to positively regulate the timing of highly patterned cell divisions (Nabel-Rosen et al., 2005). In the next stage of early mesoderm development, HOW(L) downregulates the levels of various maternal mRNAs that enable uniform mesoderm spreading over the ectoderm, an event necessary for the acquisition of specific mesodermal cell-fates at later stages (Toledano-Katchalski et al., 2007). In nematode embryos, GLD-1 levels are high only in the distal part of the gonads. GLD-1 represses the translation of maternally supplied transcripts (such as Pal-1), possibly immediately after the ribosomes have loaded on the mRNAs, to maintain the germ cell identity and block the propagation of maternal transcripts into early embryos. Also, GLD-1 simultaneously represses translation of RBP Mex through its 3′UTR, supporting Mex expression and repressive function only in the proximal part of the gonads (Mootz et al., 2004; Albarqi and Ryder, 2021).

To better understand the developmental function of Qki proteins, Hayakawa-Yano et al. (2017) performed transcriptomic profiling of *Qki* knockdown neural stem cells and revealed that the nuclear isoform Qki5 preferentially binds introns of various pre-mRNAs involved in cellular organization. Thus, Qki5 can bidirectionally control three types of alternative splicing to suppress pro-neuronal transcripts. Specifically, exon skipping occurs when Qki5 binds to the 3′ end of intronic regions immediately upstream of regulated exon, whereas exon inclusion occurs upon binding to the 5′ or 3′ end intronic regions downstream of the alternative exon. The splicing function of Qki5 was further confirmed in the *Qki* conditional KO (cKO) mouse which displayed several cellular defects. The protein γ-tubulin, which is required for microtubule nucleation from the centrosome, was mislocalized from the ventricular surface into the VZ. The authors also noticed ectopic neurogenesis, as observed by the incorrect localization of immature neurons in the VZ, and M-phase and S-phase aRG in the VZ and SVZ. These results further suggested that Qki proteins regulate cell cycle-dependent INM and inhibit neurogenesis by maintaining stemness-related genes in aRG. In particular, Qki5 positively controls the N-cadherin/β-catenin mediated adhesion, which is essential for the proper aRG polarity and preservation of the aRG ventricular surface integrity (Hayakawa-Yano et al., 2017).

To identify direct targets of Qki5 in the mouse embryonic neocortex, the same research group performed high-throughput sequencing of RNAs isolated by crosslinking immunoprecipitation (HITS-CLIP) from *Qki* cKOs (Hayakawa-Yano and Yano, 2019). The findings showed that Qki5 directly binds mRNAs coding for the Ninein protein, which is specifically localized in the aRG centrosomes. Full-length Ninein has a role in anchoring microtubules to centrosomes, which is necessary for the proper progression of INM (Shinohara et al., 2013). The full-length version of Ninein requires the inclusion of the large alternative exon 18 since it encodes the centrosome-binding protein domains. Mechanistically, Qki5 promotes the inclusion of exon 18 in the *Ninein* gene during aRG proliferation and maintenance, whereas its absence enables the exclusion of exon 18 from *Ninein*, promoting aRG-to-neuron conversion (Hayakawa-Yano and Yano, 2019). The phenotypic features of the *Qki5* cKO mouse are mostly due to the presence of the shorter *Ninein* isoform (Hayakawa-Yano et al., 2017). Overall, this evidence suggests that the nuclear Qki5 isoform is fundamental for the maintenance of the aRG self-renewal capacity during early brain development by preventing the aRG switch to neurons. Moreover, RBP-mediated alternative splicing represents a key mechanism to generate higher complexity in the neocortex.

In contrast, using HITS-CLIP from mouse postnatal forebrain, a recent study showed that the cytoplasmic isoform *Qki6* is involved in the aRG specification into an astrocyte lineage. The mechanism takes place *via* regulation of translation in peripheral astrocyte processes, possibly *via* stabilization of Qki target mRNAs, enabling their association with ribosomes (Sakers et al., 2021). Rather than binding to intronic regions, as the *Qki5* isoform does (Hayakawa-Yano et al., 2017), *Qki6* preferentially occupies 3′UTRs of a group of astrocytic mRNAs. Interestingly, the patterns of binding within 3′UTRs showed that high-affinity Qki-binding motifs are conserved and enriched near the stop codon, but also spread to the adjacent downstream regions with a lower-affinity due to the reduced presence of Qki-binding sites. This suggests that upon binding to the high-affinity sites, the tendency of Qki6 to homodimerize might enable additional bindings at lower-affinity sites, consequently facilitating mRNA looping across the stop codon which is an important event during translational elongation or termination. Utilizing “a viral approach for mosaic astrocyte-specific gene mutation with simultaneous translating RNA sequencing” (CRISPR-TRAPseq), authors elegantly revealed that Qki6 is indeed involved in an mRNA stability pathway, as seen from the reduced association of CLIP-identified targets with ribosomes in Qki cKO astrocytes. The study also showed that Qki deletion *in vivo* affects astrocyte transcriptional maturation after sorting out the subset of transcripts that had both altered ribosome-association and disturbed expression in *Qki* cKOs (Sakers et al., 2021). Another study found that the loss of RBP *Qki* from aRG has deleterious consequences at postnatal stages of neocortical development, observed from the appearance of hypomyelination with severe brain atrophy in the postnatal *Qki* cKOs (Takeuchi et al., 2020). Using *Qki* cKO *in vivo* and *in vitro* approaches, the authors showed that cytoplasmic Qki isoforms promote the fate switch from the aRG to glial precursors, which further give rise to the oligodendrocyte and astrocyte lineages. Their proposed mechanism involves stabilization of gliogenic genes upon binding of cytoplasmic Qki isoforms to specific binding sites in 3′UTRs (Takeuchi et al., 2020; Figure 9B). Taken together, three alternatively spliced isoforms of RBP Qki have major functions that span the post-transcriptional repertoire during neocortical development: while nuclear Qki5 regulates pre-mRNA splicing, Qki6 and Qki7 are mostly cytoplasmic and play important roles in mRNA stability and translation. Hence, RBP Qki is an excellent example of how regulatory functions of RBPs, and the specific type of bound mRNA targets, can evolve over time in the central nervous system.

### YTH Domain-Containing Family

**FIGURE 10:**
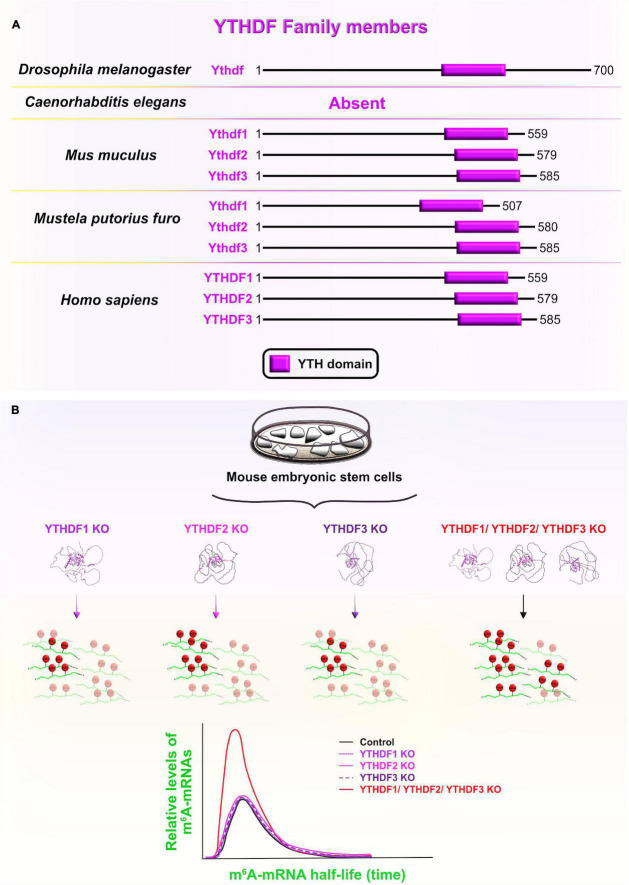
YT521-B homology domain-containing family (YTHDF) members and functional redundancy between YTHDF proteins. **(A)** Schematic presentation of RNA-binding YT521-B homology (YTH) domain (labeled in fuchsia) in evolutionarily conserved YTHDF members in *Drosophila melanogaster*, *Mus musculus*, *Mustela putorius furo*, and *Homo sapiens* consistent with UniProt database (https://www.uniprot.org). The UniProtKB accession numbers are indicated below for YTHDF protein in *D. melanogaster*: Ythdf (Q9VBZ5), *M. musculus*: Ythdf1 (P59326), Ythdf2 (Q91YT7), Ythdf3 (Q8BYK6); *M. putorius furo*: Ythdf1 (M3Y9P7), Ythdf2 (M3YVM9), Ythdf3 (M3YEM5); and *H. sapiens*: YTHDF1 (Q9BYJ9), YTHDF2 (Q9Y5A9), YTHDF3 (Q7Z739). **(B)** YTHDF paralogs (YTHDF1–3) promote N^6^-methyladenosine (m^6^A)-tagged mRNA decay in a largely redundant fashion. Only when all three cytoplasmic paralogs are deleted from mouse embryonic stem cells *in vitro*, the half-life of m^6^A-modified transcripts was significantly increased compared to non-methylated mRNAs, implying a significant decrease in the m^6^A-mediated degradation rate. This functional redundancy of the three paralogs is associated with their sequence similarities and shared mRNA target specificities. The predicted tertiary and secondary full-length protein structures of YTHDF paralogs in *Homo sapiens* are adopted from https://www.uniprot.org: YTHDF1 (Q9BYJ9); YTHDF2 (Q9Y5A9); YTHDF3 (Q7Z739).

YT521-B homology (YTH) domain-containing family proteins (YTHDF) are recognized as evolutionarily conserved RBPs across several species that specifically bind epitranscriptomic N^6^-methyladenosine (m^6^A)-containing mRNAs using their YTH domains (Figure 10A). YTHDF and YTHDC are two phylogenetic classes of YTH domains, with the former being cytoplasmic and the latter the nuclear subclass (Liao et al., 2018; Shi et al., 2019). From a developmental standpoint, the three vertebrate paralogs of the YTHDF family (YTHDF1–3) have attracted a lot of attention due to their documented role as m^6^A readers which determines the fate of m^6^A-containing transcripts during early embryogenesis (Patil et al., 2018). For example, one study has shown that the zebrafish paralog Ythdf2 is sufficient to guide the zebrafish maternal-to-zygotic transition (MZT) by inducing the reprogramming of the embryo’s transcriptome *via* global decay of the maternal mRNAs (especially the ones that were grouped together by the m^6^A tag). Indeed, deletion of *Ythdf2* from zebrafish embryos caused the retention of maternally supplied transcripts, which then caused several developmental interruptions: hampered activation of zygotic genes, delayed cell cycle progression during MZT, and delayed developmental progression through larval stages (Zhao et al., 2017).

To investigate whether the Ythdf2-dependent clearance of m^6^A-tagged transcripts is conserved during mammalian neocortical development, Li et al. (2018) ubiquitously deleted *Ythdf2* with the CRISPR/Cas9 system in mouse embryos. *Ythdf2* KOs showed delayed cortical development, as reflected in the dramatically decreased thickness of the CP and SVZ at earlier stages (E12–E14), followed by increased mortality rates at later stages of neurogenesis (E14–E18) (Li et al., 2018). Contrarily, CRISPR/Cas9-generated single *Ythdf1* or *Ythdf3* KOs did not share the same lethal destiny (Lasman et al., 2020). In *Ythdf2* KOs, impaired Ythdf2-mediated decay of neuronal related m^6^A-modified transcripts was found to be the underlying cause of retarded early cortical development, which is the stage when Ythdf2 is normally highly expressed. Aberrant transcript clearance affected the progression from symmetric to asymmetric aRG divisions at the expense of the BPs and neuronal production in the neocortex of *Ythdf2* heterozygotes, and especially *Ythdf2* KOs (Li et al., 2018).

On the other hand, Kontur et al. (2020) recently overturned the dominant role of Ythdf2 in mediating mRNA decay during zebrafish MZT. The deletion of either *Ythdf2* alone, or *Ythdf2* and *Ythdf3* together did not affect global maternal mRNA decay, the onset of zygotic genome activation and/or the developmental timing in zebrafish embryos, as proposed by Zhao et al. (2017). The authors did notice functionally redundant behavior of Ythdf proteins during zebrafish ovary development since double *Ythdf2/3* deletion prevented oogenesis and triple *Ythdf1/2/3* deletion resulted in larval lethality (Kontur et al., 2020).

In this line of thought, functional overlap at the level of m^6^A-mediated mRNA decay was recently confirmed *in vitro* in HeLa cell lines among all three YTHDF paralogs. The functional redundancy was contributed to the RBPs’ similar binding affinities and tendencies toward all m^6^A sites (Zaccara and Jaffrey, 2020). Another recent study corroborated these findings *in vivo* after systematically knocking out each of the *Ythdf* paralogs separately, or together (Lasman et al., 2020). As a result, the authors proposed context-dependent redundancy since a complete lack of *Ythdf2* cannot be compensated by other two paralogs, probably due to differences in their spatial cytoplasmic expression, while lack of either *Ythdf1* or *Ythdf3* can be functionally compensated for by the two other paralogs. The functional redundancy is also dose-dependent since a partial lack of *Ythdf2* requires the presence of at least one other reader to enable embryonic vitality. To confirm the redundancy hypothesis, the authors measured the m^6^A mRNA half-life in single or triple *Ythdf* KO mouse embryonic stem cells (ESC), and reported that their half-life is longer only in triple KO mouse ESC (Lasman et al., 2020; Figure 10B). The *Ythdf* gene thus evolved from having one copy in *Drosophila*, which directly cooperates with the RBP Fmr1 (FMRP homolog) to translationally suppress mRNAs involved in axonal growth (Worpenberg et al., 2021), to having three mammalian paralogs at high sequence identities, probably made by gene duplication events (Pervaiz et al., 2019). Afterward, the Ythdf paralogs underwent different evolutionary routes, which expanded the functional repertoire of Ythdf gene and added another regulatory layer necessary for precise control of complex events during neocortical development.

Specific YTHDF targets and the dominant role of YTHDF2 in degradation of mRNAs in an m^6^A-dependent manner and its consequences on self-renewal/differentiation have been reported in several *in vitro* studies. For example, the loss of *Ythdf2* in mouse NPC impaired their capacity to proliferate and differentiate, as seen from the appearance of abnormal neurite outgrowth (Li et al., 2018). Similarly, silencing of *Ythdf2* and *Ythdf3*, but not *Ythdf1* blocked the reprogramming of mouse embryonic fibroblasts into human-induced pluripotent stem cells (iPCSs). Since Ythdf2 and Ythdf3 are not available to recruit different deadenylase complexes, the synergistic and rapid clearance of m^6^A-modified somatic transcripts became compromised. One such target transcript is *Tead2*, which is known to impede somatic reprogramming by enabling the epithelial-to-mesenchymal transition (EMT) (Liu et al., 2020). Another study proposed the mechanism by which YTHDF2 promotes cell cycle entry through the feedforward regulatory loop with two other mediators of the cell cycle, cyclin-dependent kinase 1 (CDK1) and Wee1-like protein kinase (WEE1). In HeLa cells, the lack of *YTHDF2* reduced their proliferative capacity and caused higher accumulation of the cells which were stuck in the G2/M transition. These results suggest that CDK1 promotes YTHDF2 stability during the cell cycle, while YTHDF2 modulates the decay of m^6^A-modified WEE1 transcripts, which negatively regulate entry into mitosis (Fei et al., 2020). Also, silencing of *YTHDF2* in iPSCs phenocopies loss of pluripotency and promotes a partial acquisition of traits associated with neuronal differentiation (Heck et al., 2020). From a regulatory point of view, YTHDF2 directly binds key neuronal-specific transcripts, targets them for decay as they are produced, and keeps transcripts in a highly unstable state in iPCS. Once neuronal differentiation programs are activated, YTHDF2 levels rapidly decrease and neural-specific transcripts are allowed to achieve a new steady-state level, which in turn marks the onset of differentiation. Overall, YTHDF2 plays a pivotal role at the earliest stages of vertebrate cortical development by priming progenitors (or iPCSs) for transition into a neuronal lineage (or NPC), thereby precisely coordinating cell fate decision steps (Li et al., 2018; Heck et al., 2020).

## Implication of RNA-Binding Proteins in mRNAs Poised for Translation, Transcriptional Priming and Production of Basal Progenitors

The active transcription of pro-neurogenic genes in dividing aRG directly influences whether aRG will self-renew or differentiate into more fate-restricted progenitors or neurons (Johnson et al., 2015). In other words, aRG are transcriptionally prepatterned (or primed) (Zahr et al., 2018), and the final output expression of these fate-determining genes is regulated at the post-transcriptional level in a spatiotemporal fashion (DeBoer et al., 2014; Kraushar et al., 2014, 2015). Various RBPs (as described in detail in sections “Embryonic Lethal, Abnormal Vision-LikeELAVL and CUGBP, ELAVL-Like Family,” “Lineage Abnormal 28,” “Muscle Excess 3,” “Quaking,” and “YTH Domain-Containing Family” of this review) have been implicated in repression or derepression of pro-neurogenic transcripts in aRG, ensuring the correct timing and number of neurogenic progenies by controlling the balance between self-renewal and differentiation of aRG. Additionally, evolutionarily conserved RBPs, Smaug2 and Nanos1, represent a bimodal translational switch in which Smaug2 directly interacts and represses *Nanos1* transcripts by deporting them into repressive processing body-like granules in association with the 4E-T repression complex. Silencing of *Smaug2* triggers neuronal differentiation and hinders self-renewal by promoting aberrant translation of Nanos1 (Amadei et al., 2015). In contrast, RBP Insulin-like growth factor 2 mRNA-binding protein 1 (Imp1) protects the progenitor proliferative state by utilizing two different post-transcriptional mechanisms; Imp1 translationally represses a cohort of transcripts associated with neuronal differentiation, and simultaneously promotes mRNA stability and expression of self-renewal transcripts (such as *Hmga2*). Hence, loss of *Imp1* in the mouse neocortex caused depletion of aRG pool at the expense of their premature differentiation into BP, neurons, and glia (Nishino et al., 2013). These findings suggest the importance of transcriptional priming coupled with post-transcriptional regulation in aRG to safeguard the neuronal subtype specification at the precise time and quantity during neocortical development.

The correct and timely genesis of neurons, either directly from aRG or indirectly *via* BPs, also depends on the proper detachment of apical endfeet from the VZ; a phenomenon called neurogenic cell delamination (Kawaguchi, 2021). In both modes of neurogenesis, the type of aRG divisions thus serves as a prerequisite for cell delamination. These differentiative divisions are mostly horizontal in nature, or rarely vertical along the apico-basal axis (LaMonica et al., 2013). However, in both scenarios the neuronally fated daughter cells first inherit, then retract maternal apical endfeet in order to detach from the AJ belt in the VZ (Uzquiano et al., 2018), and migrate basally into the second germinative region, SVZ (Noctor et al., 2004; Tyler and Haydar, 2013). aRG can also utilize the asymmetric mitotic cleavage angle by oblique divisions. Obliquely dividing aRG generate one daughter cell that is destined to become either an IPC, immature neuron, or an aRG that regrows its basal fiber and remains in contact with the ventricular surface, whereas another daughter cell becomes a bRG after claiming ownership over the basal fiber, which is fundamental for the increased proliferation and maintenance of bRG (LaMonica et al., 2013; Kalebic and Huttner, 2020). Hence, different subsets of aRG can be identified based on their type of proliferative or differentiative divisions (Pinto et al., 2008). The dissimilarities in proliferative capabilities between and within progenitor types is remarkable, especially when comparing their self-renewal capacities between rodents and primates, and within the primate – underlying the importance of transcriptional priming in progenitors. This suggests that the diverse repertoire of progenitor types with hybrid transcriptional profiles during neurogenesis is responsible for the generation of progenitor heterogeneity, especially among the primates (Li et al., 2020). However, it is unclear how RBP-mediated post-transcriptional regulation of transcriptional priming drives progenitor lineage diversification in the embryonic neocortex.

To understand the molecular machinery involved in aRG to BP transition, gene expression profiling of the mouse neocortex revealed that the transcriptional factor *Insulinoma-associated protein 1* (Insm1) was specifically expressed in the subset of BP, but not in newborn neurons (Farkas et al., 2008). Insm1 promotes aRG delamination and conversion into BP by suppressing the transcription of *Plekha7*, which codes for an AJ belt-specific protein responsible for the maintenance of the aRG scaffold (Tavano et al., 2018). Another recent study showed that the RBP Elavl4 directly binds the 3′UTRs of *Insm1* transcripts and cooperates with a specific microRNA to promote their degradation in neuroblastoma cells (Kim et al., 2020). Insm1 and Elavl4 have opposite protein expression patterns early in mouse and human neocortical development: Insm1 is highly expressed in BP-genic aRG and newly generated BP, but becomes downregulated in newborn neurons (Farkas et al., 2008), whereas Elavl4 is mostly present in the CP and is absent from the VZ early in development (Popovitchenko et al., 2020). Future studies should provide a better understanding as to how this potential Elavl4-Insm1 regulatory mechanism safeguards neocortical development and the exact timing of Elavl4 in the regulation of neural progenitor delamination and production of BP.

Another AJ-related gene, *Cadherin1* (*Cdh1*), is responsible for the maintenance of AJ integrity and the aRG polarity in mouse embryos (Rasin et al., 2007), and plays a role in orienting the division axis in the *Drosophila* sensory precursor cells (Le Borgne et al., 2002). A study utilizing the ferret developing brain also showed that rapid downregulation of *Cdh1* is required for both aRG delamination and changes in the mitotic cleavage angle (from vertical to oblique or horizontal) during the narrow developmental period, shortly before the initial production of bRG. Such event sequences favor a burst generation of bRG destined for the outer SVZ (oSVZ) (Martínez-Martínez et al., 2016). This evidence suggests that post-transcriptional regulation may play a part in determining the levels of *Cdh1* transcripts during early stages of neocortical development. For example, using human colon adenocarcinoma cells, Yu et al. (2016) showed that the RBPs Celf1 and Elavl1 cooperatively modulated *Cdh1* translation by altering recruitment of *Cdh1* mRNA to processing bodies and controlling the epithelial barrier integrity. Both RBPs execute their regulatory roles by binding to different regulatory motifs in the 3′ UTRs of *Cdh1* transcripts. Celf1 acts as a *Cdh1* repressor and Elavl1 acts as a translational derepressor (Yu et al., 2016). A recent study in human prostate cancer cells implicated another RBP, hnRNPL, in the post-transcriptional regulation of *Cdh1 via* modulation of *Cdh1* transcript stability (Tan et al., 2021). Interestingly, Elavl1 directly interacts with hnRNPL in rat hepatocytes to stabilize inducible nitric oxide synthase transcripts in response to inflammatory stimuli (Matsui et al., 2008). Future research efforts should investigate whether the dynamic association of *Insm1* and *Cdh1* with either a single RBP or a group of RBPs can potentially dictate aRG fate specification, and if this mode of regulation occurs through the controlled generation of bRG in the developing neocortex.

## Implication of RNA-Binding Proteins in Expansion of the Mammalian Neocortex

In the small-brained lissencephalic mammals (e.g., rodents), IPC are the most abundant population of BP in the SVZ with restricted mitotic capacity as they typically undergo one terminal symmetric consumptive division to give rise to two immature neurons (Haubensak et al., 2004; Miyata et al., 2004; Xing et al., 2021). The remaining rare population of rodent BP belongs to bRG, which are localized more toward the upper region of the still undifferentiated SVZ (Wang et al., 2011; Vaid et al., 2018). Conversely, in large-brained gyrencephalic mammals (e.g., humans), a major population of IPC can undergo symmetric proliferative divisions, amplifying the initial progenitor pool of IPC and ultimately increasing the number of postmitotic neurons (Kriegstein et al., 2006). The evolution of primates is specifically characterized by the impressive expansion in the number, complexity, and variety of bRG, which are thought to be instrumental in the vast differences of neuronal abundance and the overall size of the neocortex between different species (Fietz et al., 2010; Reillo et al., 2011; Li et al., 2020). There are several reasons to suspect that both evolutionary conservation and remarkable diversification of RBP regulatory function strongly contribute to the accelerated evolutionary events that built the complexity of progenitor-primed state in primates. The region-specific and cell subtype-specific expression of RBPs (McKee et al., 2005; Bedogni and Hevner, 2021), together with autoregulative, cooperative, and competitive behaviors between RBP point to the existence of master regulatory units (termed RBP chains and RBP–RBP networks) that can delicately modulate the expression of a wide set of mRNA targets (Quattrone and Dassi, 2019). Hence, the growing number of RBPs and the gradual evolution of their regulatory signatures may partially explain how post-transcriptional regulation is implicated in neocortical expansion.

**FIGURE 11:**
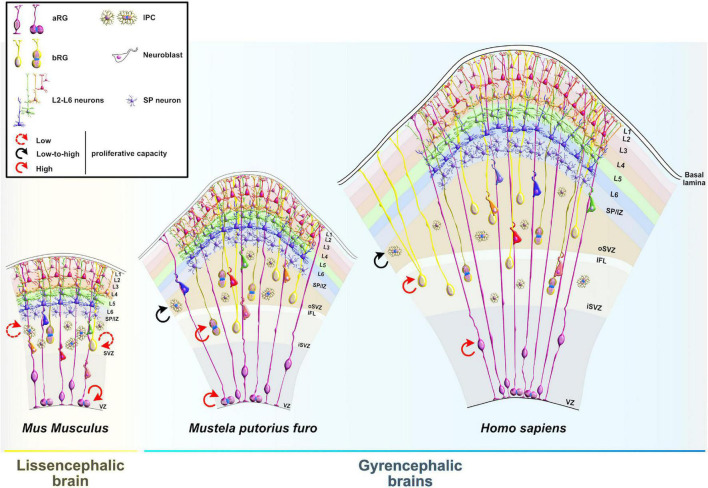
Expansion of the neocortex. Mice have smooth and small (lissencephalic) neocortex, whereas ferrets and humans have folded and expanded (gyrencephalic) neocortex. The evolutionary expansion of the neocortex and vast species–species differences are linked to the expansion and specialization of the subventricular zone (SVZ) into inner SVZ and outer SVZ, accompanied with the substantial expansion of the basal progenitors, particularly basal radial glia. Although apical radial glia have high proliferative capacities across species (red arrow), the evolution of basal radial glia is associated with the impressive increase in their number, complexity, variety, and proliferative capacities (red arrow) in gyrencephalic species. In lissencephalic brains, intermediate progenitor cells represent the largest proportion of basal progenitors and, like basal radial glia, have low proliferative capacity (broken red arrow). In gyrencephalic brains, intermediate progenitor cells have lower relative abundance compared to basal radial glia and are characterized by a wide range of proliferative capacities (black arrow). aRG, apical radial glia; IPC, intermediate progenitor cell; bRG, basal radial glia; VZ, ventricular zone; SVZ, subventricular zone; iSVZ, inner SVZ; IFL, inner fiber layer; oSVZ, outer SVZ; SP/IZ, subplate/intermediate zone; L1–L6, layers 1–6.

As a result of the proliferative explosion of BP, the extensively enlarged SVZ becomes subdivided into the inner-SVZ (iSVZ) and oSVZ. The iSVZ of gyrencephalic mammals is composed mostly of the IPC population during neurogenesis, and it is comparable in its thickness to the still undifferentiated SVZ of the lissencephalic mammal. However, the primate oSVZ is the largest proliferative compartment due to the presence of highly proliferative and neurogenic bRG, which tend to use the available area of the thickening SVZ for symmetric proliferative (two daughter bRG) or asymmetric proliferative divisions (one daughter bRG with self-renewal capacity, and another daughter IPC or neuron) (Smart, 2002; Kriegstein et al., 2006; Fietz et al., 2010; Silbereis et al., 2016; Xing et al., 2021). While the cell cycle kinetics of the lissencephalic progenitors generally decrease overtime (Takahashi et al., 1995), the peak expansion of superficial-layer neurons occurs during the later stages of primate neurogenesis in tandem with the upsurge of bRG proliferation (Figure 11), as well as the transformation of aRG into more truncated morphologies. aRG cease being a main scaffold, guiding the migration of newborn neurons since their basal processes terminate in oSVZ. As proposed in the “Supragranular Cortex Expansion Hypothesis,” superficial-layer neurons can only reach their final destination by climbing along bRG basal fibers, which are, now, a part of a discontinuous scaffold made by truncated aRG and different bRG morphotypes (Nowakowski et al., 2016; Kalebic and Huttner, 2020). The prenatal neurogenesis is finalized once aRG and BP begin symmetric or asymmetric consumptative divisions, giving rise to postmitotic neurons. The progenitors then shift to gliogenesis, which is a process that completely depletes the progenitor pool by generating astrocytes and oligodendrocytes (Xing et al., 2021).

**FIGURE 12:**
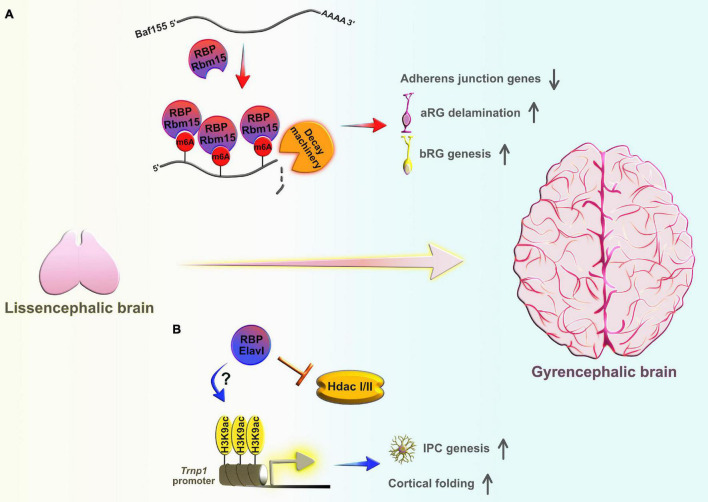
The contribution of RNA-binding proteins (RBPs) to the production of basal progenitors and expansion of the neocortex. **(A)** RBP Rbm15 regulates epitranscriptomically the expression of the chromatin remodeler *Baf155*, which in turn controls the expression programs of genes encoding AJ proteins. As a part of the N^6^-methyladenosine (m^6^A) methyltransferase complex, Rbm15 initiates the addition m^6^A modification *Baf155* mRNA, and governs its decay through the mRNA methylation machinery. The downstream effect of the reduced BAF155 mRNA stability causes downregulation of AJ proteins at the ventricular surface, supporting the generation of basal radial glia (bRG), initially dependent on apical radial glia (aRG) delamination during early stages of neocortical development. **(B)** At later stages of neocortical development, another epigenetic mechanism, H3 lysine 9 acetylation (H3K9ac), drives the expansion of the neocortex by regulating the expression of regulatory factors (e.g., *Trnp1*) which are, in turn, responsible for the incremental basal progenitors’ self-renewal capacity. As such, inhibition of class I/II histone deacetylases (Hdac I/II), and/or H3K9ac epigenome editing of *Trnp1* promoter was sufficient to promote the genesis of intermediate progenitor cells (IPC) and evoke cortical folding in mice. Elavl family members can block Hdac II activity. These RBPs may act as upstream regulators of local histone hyperacetylation, boosting neuronal output and inducing gyrification during neocortical evolution.

All types of BP are recognized as key players in the expansion and complexification of the primate brain due to their capacity to proliferate by symmetric divisions. The post-transcriptional regulation of BP proliferation and specification is essential for proper neocortical development. Recent efforts have tried to illuminate the contribution of RBP in neocortical expansion especially because they are engaged in almost every step of post-transcriptional regulation. One such RBP, RNA-binding motif protein 15 (Rbm15), regulates the delamination of cortical progenitors by promoting degradation of *BRG1 Associated Factors* (*BAF*) *155* (*Baf155*) *via* m^6^A RNA methylation machinery (Figure 12A). The repressive function of Rbm15 is reflected in the opposite expression pattern relative to its target *BAF155* during mid-neocortical development; low levels of Rbm15 and high levels of Baf155 are present in the VZ and SVZ, whereas the opposite is true for the IZ and CP (Xie et al., 2019). Narayanan et al. (2018) showed that Baf155, an integral component of the chromatin remodeling complex BAF, facilitates the genesis of BP by controlling their gene expression programs. The mouse *Baf155* cKO embryos exhibited aberrant development: an increased pool size of bRG as a result of a substantially reduced number of IPC and VZ-located aRG, disrupted delamination with ectopic dispersion of both aRG and bRG-like cells, and a change in the orientation of the mitotic cleavage planes from vertical to horizontal or oblique (Narayanan et al., 2018). Similarly, overexpression of *Rbm15* in mouse embryos repressed *Baf155* expression, and recapitulated the phenotypic consequences observed in *Baf155* cKOs, including a defect in the formation of AJ and abnormal delamination of cortical progenitors, both of which are the hallmarks of bRG genesis under normal conditions (Xie et al., 2019). Some genes enriched in human bRG are upregulated in *Baf155* cKOs. Future studies should elucidate the extent to which Rbm15, an upstream regulator of *Baf155*, contributes to progenitor function and bRG production.

Recently, H3 lysine 9 acetylation (H3K9ac) was identified as another epigenomic mark relevant for BP expansion and folding of the primate neocortex. Using mass spectrometry to detect differences in the epigenetic landscapes between the mouse and human developing neocortices, Kerimoglu et al. (2021) revealed that mouse BP have substantially lower H3K9ac levels relative to human BP. The chemical inhibition of class I/II histone deacetylases (Hdac I/II), an enzyme that removes acetyl groups, in *Baf155* cKOs promoted the proliferation burst of various BP types and boosted their proliferative capacities. Remarkably, the authors also noticed that elimination of Hdac I/II activity stimulated the gyrification of the mouse lissencephalic brain. This study revealed that BP amplification is mediated through the activation of *Trnp1* expression. Histone H3K9ac epigenome editing of the *Trnp1* promotor specifically increased the production of IPC and distinct neuronal subtypes and induced *de novo* cortical folding in the mouse neocortex (Kerimoglu et al., 2021). Even though the upstream regulators of the H3K9ac mechanism responsible for the enhanced production of BP are yet to be identified, RBPs have already been recognized as bona-fide epigenetic regulators of gene expression. This revelation challenges the conventional view that RBPs predominately regulate gene expression at the post-transcriptional level (Tan and Yeo, 2016; Du and Xiao, 2020; Kosti et al., 2020). Interestingly, Elavl family members can promote local histone hyperacetylation by directly blocking Hdac II activity, inducing higher transcription elongation rates (Zhou et al., 2011). It is reasonable to suggest that RBPs (Elavl members particularly) may act as epigenetic modulators, indirectly repressing or derepressing the expression of genes involved in the rapid expansion of the primate neocortex (Figure 12B).

A recent study proposed that neocortical expansion heavily depends on the initial production of the NE, much before the acquisition of neurogenic aRG identity. The larger size of human organoids relative to those from other apes prompted Benito-Kwiecinski et al. (2021) to focus on identifying species–species differences at the level of apical progenitors by using cerebral organoids from human, gorilla, and chimpanzee iPCS-derived cells. While an apically constricted transition from NE to aRG in rodents typically lasts for a couple of hours, the primate transition is characterized first by the prolonged morphological rearrangements of progenitors which occur over a course of several days in ape organoids and lasts even longer in human organoids. This EMT-like transition includes a novel morphological state of NE, called transitioning NE (tNE). Interestingly, the NE-to-tNE transition happens in tandem with the slowing down of the cell cycle, which possibly influences the proliferative capabilities of human progenitors. When compared to apes, the delayed maturation into tNE in humans allows for more time for the NE to proliferate and proportionally increase the progenitor pool and final neuronal output. This event parallels with the timing of ZEB2 expression, which is an EMT-related transcription factor. Remarkably, premature expressions of ZEB2 in human organoids phenocopies the earlier expression of tNE, which is typically observed in ape organoids (Benito-Kwiecinski et al., 2021). Previous oncological studies have uncovered the role of RBPs in the regulation of *ZEB2* mRNAs metabolism. For example, RBP hnRNP C partners with specific lncRNA in order to directly promote *ZEB2* mRNA stability, which results in increased EMT progression and cell migration (Zhang et al., 2019). Similarly, the RBP Elavl1 positively regulates the progression of the EMT transition by increasing the expression of 3′UTR-bound *ZEB2* transcripts through translation or stability (Prislei et al., 2015). Even though the RBP-ZEB2 regulatory network has been formulated in the context of cancer progression, it still provides useful guidance for future studies which may reveal the identity of the critical RBPs players behind the modulation of ZEB2 metabolism and the evolutionary shaping of primate neocortex.

## Establishment of the Unique Layered Structure of the Neocortex

Next to the adaptations in the progenitor cell cycle kinetics, neuronal migration is also considered a pivotal mechanism underlying neocortical evolution. The migration of cortical neurons is a multievent and a tightly regulated feature of neocortical development during which newborn postmitotic neurons sense the environmental cues and convey the received information into tightly orchestrated reorganizations of their cytoskeleton. Such dynamic behavior is partially controlled *via* RBPs at the post-transcriptional level (Krsnik et al., 2020). Depending on the neuronal place of birth and neocortical thickness at the distinct stages during neurogenesis, neuronal migration can be distinguished by four different types of movements: (1) somal translocation, (2) multipolar migration, (3) glial-guided locomotion, and (4) terminal somal translocation (Heng et al., 2010; Krsnik et al., 2020). All these types of cellular motility are equally important for the generation of six neocortical layers. Radial migration begins with neuronal delamination during which earliest-born neurons in the VZ detach their apical endfeet from the AJ belt (Kon et al., 2017; Arimura et al., 2020). As a result, the delaminated neurons acquire bipolar morphology and start their premigratory locomotion, called somal translocation (Nadarajah et al., 2001). Hence, the earliest-born neurons first extend their basal processes to reach the pial surface, followed by process retraction that pulls cell bodies into a newly formed transient zone called the preplate (the primordial plexiform layer). Subsequently, another wave of early neurons migrates out of the VZ to form the CP by reaching and splitting the preplate into two more transient layers called: the MZ and the SP. The earliest-born neurons that remain in the MZ (defined as layer I in the future six-layered neocortex) will later differentiate into Cajal–Retzius neurons that secrete Reelin, a stop signal for migrating neurons (D’Arcangelo et al., 1995; Boyle et al., 2011).

At later neurogenic stages, the CP expands both radially and tangentially, which is why neurons generated by progenitors from both VZ and SVZ start implementing different modes of migration. Upon arrival to the SVZ, neurons first dramatically rearrange their morphology from bipolar to transient multipolar identity (Noctor et al., 2004; LoTurco and Bai, 2006). It is suggested that bipolar–multipolar transition, followed by brief multipolar migration in the SVZ supports the horizontal neuronal spread to institute functionally relevant cortical column circuits (Tabata and Nakajima, 2003; Cooper, 2014). Then, later-born neurons convert back to bipolar morphology to resume their directional migration toward the CP (Shu et al., 2004; Tabata et al., 2013). Even though our knowledge about processes regulating multipolar–bipolar transition is still limited, some of these processes are post-transcriptionally regulated by RBP, such as FMRP (La Fata et al., 2014) and PIWI1 (Zhao et al., 2015). Recently, Liu et al. (2019) implicated another RBP, called non-POU domain-containing octamer-binding protein (NONO), in the tight control of neuronal migration, partly *via* modulating the expression of vitronectin which is an extracellular matrix protein known to promote cell adhesion and neuritogenesis (Katic et al., 2014). NONO OE in the mouse embryonic neocortex resulted in impaired multipolar–bipolar transition and neuronal polarity, which in turn delayed the directional migration and morphological maturation of late-born neurons in the developing neocortex (Liu et al., 2019). Conversely, a recent *ex vivo* study demonstrated that the premigratory multipolar phase, normally found in rodents, is extremely rare during macaque cortical development. Instead, the vast majority of macaque premigratory neurons exhibit bipolar morphologies, inherited from the mother progenitors (Cortay et al., 2020). Bipolar neuronal progeny exhibits a high degree of flexibility by rapidly extending and retracting their processes. Such event sequences not only optimize the initial step of radial migration, but also enable a swift decision regarding the direction of neuronal migration, the essential trait for tangential dispersion and lateral expansion of the primate neocortex (Kalebic and Namba, 2021). It would be interesting to examine if these early premigratory morphotypes in macaque embryos are possibly under post-transcriptional regulation by RBPs. One potential candidate might be the RBP Unkempt, which has been previously recognized as a master translational regulator of early neuronal morphology in mouse embryos. Unkempt acts as an upstream translational modifier of various mRNA targets, some of which code for other RBPs, which, in turn, translationally control neuronal morphology programs during neocortical development. When ectopically expressed, Unkempt also has the ability to polarize cells of non-neuronal origin, bolstering its supremacy in a hierarchical RNA regulon (Murn et al., 2015).

The journey of directional migration is quite challenging since neurons must first find their way through the axon-rich IZ, then they must pass through the transient compartment SP until they finally reach their destination within the CP, which is already densely packed due to the prior arrival of older-born neurons (Smart, 2002; Kostović, 2020; Kostović et al., 2021). To reach their destined position in the CP, neurons travel along radial glial basal fibers, a migration mode known as glial-guided locomotion (Rakic, 1972; Nadarajah et al., 2001). Once they arrive to the uppermost layer of CP, neurons stop their radial migration and adopt terminal somal translocation as their final type of glial-independent movement, in which the cell body quickly moves a short distance upward to localize just beneath the MZ (Nadarajah et al., 2001; Sekine et al., 2011). Again, RBPs appear to be major players in regulating the later steps of neuronal migration. Zhao et al. (2020) showed that the deletion of RBP *Elavl1* in post-mitotic neurons affects F-actin dynamics, which translates into the delayed cell motility of later-born neurons without any effect on the multipolar–bipolar transition or neuronal polarization. As a result, *Elavl1* cKO later-born neurons preferentially localized in the deeper, instead of the upper, cortical layers in the mouse embryonic neocortices. The mechanism partially takes place through the stabilization of F-actin modulator, *Profilin1* mRNA, upon Elavl1 binding to its 3′UTRs. OE of *Profilin1* in *Elavl1* cKOs neurons thus rescued the migratory phenotype (Zhao et al., 2020). The loss of another RBP, *RNA-binding motif 4* (*Rbm4*), results in elevated levels of *Disabled-1* (*Dab-1*) isoform lacking tyrosine-encoding exons 7/8, which causes a severe migratory deficit and aberrant positioning of the later-born neurons in the mouse embryos. The isoform-specific alternative splicing of *Dab-1*, which is known to be essential for proper neuronal migration, is accomplished through dynamic competition between Rbm4 and another RBP, polypyrimidine tract-binding protein 1 (Ptbp1). While Ptbp1 promotes exclusion of exons 7/8 early during neocortical development, Rbm4 governs their inclusion during mid developmental stages, generating a full-length *Dab1* isoform that predominates during migration of later-born neurons. This splicing switch enables phosphorylation of the tyrosine residues of exons 7/8 in response to Reelin signaling, which subsequently activates cytoskeletal machinery and coordinates directional migration (Dhananjaya et al., 2018).

Overall, radial migration is considered complete when migratory neurons are precisely organized in the CP forming six distinct layers, with the youngest neurons continually layering on top of the oldest. Thus, early-born neurons are responsible for the generation of deeper (older) layers (layers V and VI), while later-born neurons are destined for superficial (younger) layers (layers II–IV) (Rakic, 1974). Importantly, neurons are born from progenitors that are already programmed with a map directing the newborn neurons to a specific location in the CP, as designed by the map from the mother progenitor cell. The specific targeted location of cortical neurons is contingent upon their horizontal (tangential) and vertical (radial) coordinates. The former of the two is established with respect to the positioning of the mother progenitors in the VZ and relates to the specific function the neurons will carry in the brain area, whereas the latter is determined by time of birth in the neocortex and responds to the acquisition of the subtype-specific fates (Rakic, 1988; Dehay et al., 1993, 2015; Klingler et al., 2019; Heavner et al., 2020). Once properly integrated in the neocortex, neurons start making excitatory and inhibitory synapses, which are a prerequisite to the formation and wiring of early neuronal circuits. One complex function of the cortex’s early neural circuit entails the sending of axons to their targets, as directed by cortical neurons, an act by which permits the connection of neurons in other cortical and also subcortical regions (Jabaudon, 2017). Not surprisingly, various RBPs, including Arpp21, Rbfox1, Lin28, Pumilio2, Staufen2, Elavl4, Ythdf, and FMRP, regulate the expression of mRNAs involved in synaptic transmission and neurite outgrowth. This directly serves as a link between RBPs, neuronal dysfunction, and occurrence of neurodevelopmental disorders (Rehfeld et al., 2018; Vuong et al., 2018; Jang et al., 2019; Bowles et al., 2021; Schieweck et al., 2021; Sena et al., 2021; Worpenberg et al., 2021).

## Conclusion

The progression of cortical neurogenesis can be succinctly understood *via* the concept of progenitor temporal patterning (Bayraktar and Doe, 2013). This concept explains how the timely expression of transcripts alters progenitor fate; the effect of the patterning is the diversification of neuronal types and a dramatic expansion of the mammalian neocortex (Arendt et al., 2019).

Post-transcriptional regulation guided by RBPs is one of the regulatory mechanisms that shapes the final output of progenitor temporal patterning. RBPs can contain either one or multiple RNA-binding domains (RRM, KH, YTH, CSD, etc.) which participate in the binding of available transcripts, thus regulating at least one of the many aspects of transcripts’ life cycles in progenitors and their neuronal and glial progeny. RBP, which are highly evolutionarily conserved, have also acquired structural and functional adaptations to regulate RNA metabolism with vigilant coordination. Their function has expanded from nuclear splicing, polyadenylation, RNA editing, and reading of epitranscriptomic modifications to cytoplasmic transport, stability, localization, and translation. All of these functions put RBP in a unique position to influence progenitor proliferation, neuronal differentiation, migration, and neuronal operating capacity (Kraushar et al., 2014; Yang et al., 2014; Amadei et al., 2015; Zahr et al., 2019; Popovitchenko et al., 2020; Seo and Kleiner, 2021).

It may be insufficient to classify RBPs solely by their conserved RNA-binding domains; they are a much more heterogeneous group than previously thought (Gerstberger et al., 2014). Moreover, recent studies have identified new RNA-binding regions in proteins involved in metabolic and enzymatic pathways which lack conserved RNA-binding domains but still moonlight as RBPs, influencing the destiny of transcripts (Kwon et al., 2013; Hentze et al., 2018; Nechay and Kleiner, 2020). The mRNA targets and mechanisms by which these unconventional RBPs influence progenitor fate, including their degree of importance in neocortical neurogenesis, remain to be elucidated. RNA regulons represent another control layer that enables the convergence of different post-transcriptional mechanisms to effectively regulate the destiny of functionally related transcripts (Simone and Keene, 2013), ultimately influencing cell cycle progression, neuronal migration, or specification. This mechanism comes in handy, for example, to rapidly activate the pro-neuronal transcripts in neurons, which were inherited upon differentiation from transcriptionally primed aRG, where the same transcripts are translationally repressed or degraded *via* the post-transcriptional mechanism (Yang et al., 2014; Telley et al., 2019).

Apart from the RNA-binding domains, other features strongly contribute to the RBP functional output. The protein structures which RBP use to bind their targets are also extremely diverse (e.g., hydrogen bonds, Van der Waals, hydrophobic, and π interactions). A handful of evolutionarily conserved RBPs have been thoroughly categorized according to the type of RNA-binding domains they possess and the mechanism of RBP–mRNA interactions (Corley et al., 2020). It is possible that the diversification of domains’ specific binding strategies has partially led to the expansion of the RBP functional repertoire. All the RBP mentioned in this review, together with many others such as Staufen (Heraud-Farlow and Kiebler, 2014) and Pumilo (Nishanth and Simon, 2020) play various fundamental roles which extend beyond the embryo to neurogenesis and synaptic establishment and transmission. Because precise regulation of gene expression depends heavily on the intramolecular interactions between RBP(s) and their target(s), there is a great need to systematically investigate a vast number of yet unexplored RBPs to understand their regulatory properties, especially within the context of the dynamic changes during neurogenesis. Furthermore, identification of mRNA target(s) of RBPs in combination with increasing knowledge of RBP–mRNA interactions dynamics may advance the accuracy of emerging RBP-targeting therapies in the brain (Jackson and Kochanek, 2020).

It is also possible that mammals, especially primates, acquired more efficient RBP-driven mechanisms to respond promptly and adequately to the broader functional demands of the apical and BPs, which arose due to the expansion and changes in composition of progenitors during neocortical evolution. This can partially be explained by the fact that RBPs rarely act alone. They rather interact directly or indirectly with each other to synergistically enhance the binding affinity to their targets. Multifunctionality of RBPs can also be achieved through graded combinatorial interplay at multiple hierarchical levels where different RBPs enhance, diminish, overrule, or autoregulate the function of each other. Such a complex network of RBP–RBP interactions is required to fine-tune various cellular events during all stages of neurogenesis (Dassi, 2017). It is, thus, of utmost importance to clearly define, at a single-cell level with genetic lineage tracking, how RBP-specific mechanisms contribute to neuronal diversity and differences in brain architecture (Feng et al., 2021). A comprehensive understanding of RBPs’ features, including RNA-binding domain architecture, RBP–mRNA interactions, and RBP–RBP interactions, may illuminate how exactly RBPs regulate transcript expression in progenitors and neurons, which will, in turn, elucidate the complex cellular mechanisms underlying neocortical development and neurodevelopmental diseases.

## Author Contributions

IS and M-RR contributed to conception and design of the manuscript. IS wrote the first draft and figures, and introduced changes. Both authors contributed to manuscript revision, read, and approved the submitted version.

## Conflict of Interest

The authors declare that the research was conducted in the absence of any commercial or financial relationships that could be construed as a potential conflict of interest.

## Publisher’s Note

All claims expressed in this article are solely those of the authors and do not necessarily represent those of their affiliated organizations, or those of the publisher, the editors and the reviewers. Any product that may be evaluated in this article, or claim that may be made by its manufacturer, is not guaranteed or endorsed by the publisher.
